# Tumoral expression of drug and xenobiotic metabolizing enzymes in breast cancer patients of different ethnicities with implications to personalized medicine

**DOI:** 10.1038/s41598-017-04250-2

**Published:** 2017-07-06

**Authors:** Yan Li, Albert Steppi, Yidong Zhou, Feng Mao, Philip Craig Miller, Max M. He, Tingting Zhao, Qiang Sun, Jinfeng Zhang

**Affiliations:** 1 Department of Breast Surgery, Peking Union Medical College Hospital, Peking Union Medical College, Chinese Academy of Medical Sciences, Beijing, China; 2 Department of Statistics, Florida State University, Tallahassee, FL 32306 USA; 3 University of Miami Miller School of Medicine, Sylvester Comprehensive Cancer Center, Miami, FL 33136 USA; 4 Center for Human Genetics, Marshfield Clinic Research Foundation, Marshfield, WI 54449 USA; 5 Biomedical Informatics Research Center, Marshfield Clinic Research Foundation, Marshfield, WI 54449 USA; 6 Department of Geography, Florida State University, Tallahassee, FL 32306 USA

## Abstract

Drug and xenobiotic metabolizing enzymes (DXME) play important roles in drug responses and carcinogenesis. Recent studies have found that expression of DXME in cancer cells significantly affects drug clearance and the onset of drug resistance. In this study we compared the expression of DXME in breast tumor tissue samples from patients representing three ethnic groups: Caucasian Americans (CA), African Americans (AA), and Asian Americans (AS). We further combined DXME gene expression data with eQTL data from the GTEx project and with allele frequency data from the 1000 Genomes project to identify SNPs that may be associated with differential expression of DXME genes. We identified substantial differences among CA, AA, and AS populations in the expression of DXME genes and in activation of pathways involved in drug metabolism, including those involved in metabolizing chemotherapy drugs that are commonly used in the treatment of breast cancer. These data suggest that differential expression of DXME may associate with health disparities in breast cancer outcomes observed among these three ethnic groups. Our study suggests that development of personalized treatment strategies for breast cancer patients could be improved by considering both germline genotypes and tumor specific mutations and expression profiles related to DXME genes.

## Introduction

Breast cancer (BRCA) is a heterogeneous disease and understanding this heterogeneity is a key challenge in the development of effective personalized treatment strategies. Individual breast cancer patients with pathologically similar tumors may respond very differently to the same standard treatments; the mechanisms for this disparity are not well understood. The incidence and death rates of breast cancer differ among people from different ethnic groups. From 2008–2012, the incidence rates of breast cancer per 100,000 people were 128.1, 124.3, and 88.3 for non-Hispanic white, non-Hispanic black, and Asian and Pacific islander populations, respectively^1^. Additionally, the breast cancer specific death rates per 100,000 people are 21.9, 31.0, and 11.4 for non-Hispanic white, non-Hispanic black, and Asian and Pacific islander, respectively^1, 2^. In addition to differences in socioeconomic status and lifestyle^3–5^, genetic factors likely are critical in establishing breast cancer health disparities among these three ethnic groups^1, 6–11^, and identifying these genetic factors will lead to development of more effective personalized medicine. Drug and xenobiotic metabolizing enzymes (DXME) play important roles in patients’ responses to treatment and in the development of drug resistance.

The study of DXME has generally focused on enzyme activities in the liver or blood, and association studies have mostly examined germline variants in DXME coding regions from patients. In recent years it has been reported that intratumoral expression of DXME significantly affects tumor drug response and the onset of resistance to therapy, and it has been suggested that drug concentrations in plasma or tissues alone cannot completely explain the efficacy of drugs in target organs or tumor tissues. Although variability of drug metabolism in the liver must be considered as a potential factor mediating drug sensitivity or resistance, intracellular penetration, accumulation, distribution, metabolism, and elimination are important parameters governing the efficacy of drugs that interact with targets localized within cancer cells^12^. For example, induction of cytochrome P450 (CYP) activity may facilitate the onset of drug resistance by accelerating the degradation and clearance of anti-cancer agents in cancer cells^13^.

In solid tumors, the extracellular and intracellular distribution of drugs exhibits a high degree of variability, and is largely controlled by DXME and influx and efflux systems that transport drugs into and out from cells. Expression of DXME within tumor cells is known to play a role in tumor cell survival and in tumor-specific absorption, distribution, metabolism, and excretion (ADME) of drugs^14^. In cancer subclones, there tends to be a strong genomic instability that leads to highly variable expression of DXME. Cancer cell drug resistance or sensitivity is critically impacted by expression of DMXE within tumors, and understanding which specific DXME contribute to response to particular drugs will lead to better precision medicine^12, 15^.

Patient ethnicity may influence differences in the pharmacokinetics (PK) and pharmacodynamics (PD) of drugs, resulting in variability in responses to drug therapy and contributing to ethnic disparities in patient outcomes^16^. A previous study had profiled the expression of 21 CYP family genes in 170 breast tumor tissues and found that the expression of several CYP family genes was correlated with tumor grade, molecular subtype, or patient survival^17^. By comparing DXME gene expression, pathway activation, and associated genotypes in breast cancers of patients from different ethnic backgrounds, we could gain important insight on the variation of these enzymes in diverse patient populations, which would provide useful guidance in the development of precision treatment strategies.

In this study we used breast cancer gene expression data obtained from The Cancer Genome Atlas (TCGA) to study the expression patterns of DXME across Americans from three different ethnic backgrounds: Caucasian American (CA), African American (AA), and Asian American (AS). We combined gene expression data with eQTL data from the Genotype-Tissue Expression (GTEx) Project and genotype information from the 1000 Genomes Project to perform an integrative study to identify Single-nucleotide polymorphisms (SNPs) that may associate with differential DXME gene expression between these different racial groups. This approach not only identified SNPs potentially associated with DXME expression, but also provided valuable insight into the mechanisms by which gene expression links genotype (different allele frequencies in different ethnic groups) and phenotype (different incidence and death rates among different ethnic groups).

This current study may help researchers better understand the biological factors causing the health disparity among diverse ethnic groups, while also improving understanding of breast cancer heterogeneity through studying the expression of DXME. Characterizing differential expression and activity of DXME in breast tumors from a diverse patient population may identify novel factors and mechanisms that underlie ethnic disparities in breast cancer outcomes.

## Results

### Drug and xenobiotics metabolizing enzymes (DXME)

Names and gene symbols for DXME genes were obtained from several KEGG pathways, including “drug metabolism - cytochrome P450”, “metabolism of xenobiotics by cytochrome P450”, and “drug metabolism - other enzymes”^18–20^. In total, 88 genes representing 13 enzyme classes were identified (Supplementary Material). Among these 88 genes, there are 24 cytochrome P450 (CYP family) genes.

### Differential expression of DXME across CA, AS, and AA breast cancers

**Figure 1:**
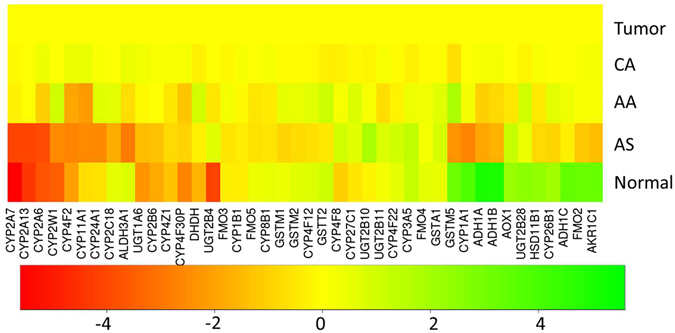
The expression of 42 drug and xenobiotics metabolizing enzymes across breast cancers for Caucasian American (CA), African American (AA), Asian American (AS), all BRCA cancer samples from the three races (Tumor), and all normal samples from the three races (Normal). The mean expressions of all tumor BRCA samples (Tumor) were used as a reference and relative mean expression values from other groups were plotted using a color scheme to show qualitative differences. The values represent fold changes of different groups vs. Tumor group. There are totally 42 DXME genes that display significant differential expression between at least two comparisons (Tables 1 and S2, fold change ≥ 2 and adjusted p-value ≤ 0.05).

Differential expression of DXME genes among the CA, AS, and AA breast cancers was analyzed using DESeq2 (Fig. 1). The mean expressions in CA BRCA were used as a reference and relative mean expression values from AA and AS BRCA were plotted using a color scheme to show qualitative differences. The genes in Fig. 1 represent 42 DXME genes that display significant differential expression between at least two ethnic groups (fold change ≥ 2.0 and adjusted p-value ≤ 0.05). The expression differences for many of the genes across the three ethnicities are quite large, which is consistent with previous observations of a large number of genetic variants in these genes^21–26^. It is noteworthy that AS BRCA have overall lower expressions for many DXME genes compared to either CA or AA BRCA. The number of DXME genes that exhibited higher expression in AA compared to CA BRCA was similar to the number of DXME genes that exhibited lower expression in AA compared to CA BRCA (Fig. 1). Overall, the greatest differential expression of DXME genes was seen in AA vs. AS BRCA, followed by the differences between AS and CA BRCA. Expression of DXME genes was the most similar between AA and CA BRCA.

Differential expression of CYP genes was also compared between different stages of BRCA and different molecular subtypes. Due to limitation in sample size, stage I and II are combined to form an early stage (ES) group and stage III and IV are combined to form a late stage (LS) group. Expression of CYP genes was compared between ethnic groups (CA vs. AA; CA vs. AS; AA vs. AS) and by tumor stage and molecular subtype (Table 1). Below we discuss a few genes in more detail.

**Table 1:** Significantly differentially expressed (fold change >= 2 and adjusted p-value <= 0.05) CYP genes in various comparisons.

Gene	CA vs AA	CA vs AS	AA vs AS	Metabolism Substrates	Polymorphic
CYP1A1		ES	ES	Toremifene, Tamoxifen, Imatinib, Flutamide, Benzo pyrene	Yes
CYP1B1	TN			Tegafur, Docetaxel, Flutamide	Yes
CYP2A6	ES, LS, All	All, LA	All, ES	Cyclophosphamide, Ifosfamide, Fluorouracil, Nicotine, Aflatoxin	Yes
CYP2A7	ES, LA, All		All, ES, LA		
CYP2A13			All, ES	Nicotine, Aflatoxin	Yes
CYP2B6	LS			Tamoxifen, Cyclophosphamide, Ifosfamide	Yes
CYP2C18	LS		All, ES		
CYP27C1	LS				
CYP2W1			All, ES		
CYP3A5		All		Ifosfamide, Tamoxifen, Paclitaxel	Yes
CYP4F2			LA		Yes
CYP4F8			TN		
CYP4F12	LA				
CYP4F22		ES	ES		
CYP4F30P	ES, LA, All				
CYP4Z1	ES, LA, All				
CYP26B1			All, ES, TN		
CYP8B1		All, ES, LA	All, ES		
CYP11A1			LS		
CYP24A1	LA	All, ES, LA	ES		

For all the other DXME genes, see Supplementary material (Table S2). Column 2, 3 and 4 are for comparison between CA and AA, CA and AS, and AA and AS, respectively. In column 2, 3 and 4, we list the comparisons where the gene is significantly differentially expressed. All: overall comparison; ES: early stage (stage I, II); LS: late stage (stage III, IV); LA: luminal A; LB: luminal B; H2: HER2; TN: triple-negative. An ES in column CA vs. AS for gene CYP1A1 means CYP1A1 is significantly differentially expressed between early stage breast cancer in CA and early stage breast cancer in AS.

#### CYP4Z1

The mean expression (in reads per million, or RPM) of CYP4Z1 in adjacent normal tissue (all matched normal tissue samples at TCGA combined), BRCA tissue (all racial groups combined), and separately for CA, AS, and AA breast tumor tissue was 36.9, 92.2, 101, 52.7, and 63.6, respectively. Expression of CYP4Z1 in normal breast tissue was lower than in breast tumor tissue; among the three ethnic groups, AS breast cancer displays the lowest expression of this gene. While the substrate of CYP4Z1 is unknown, CYP4Z1 mRNA was detected in breast carcinoma tissue and in normal mammary gland tissue, whereas only marginal expression was found in all other tissues^27^. Additionally, it was reported that expression of the CYP4Z1 gene is upregulated by activated glucocorticoid and progesterone receptors^28^. Overexpression of CYP4Z1 is associated with increased production of 20-hydroxyeicosatetraenoic acid (20-HETE) in BRCA, and it has been hypothesized that CYP4Z1 metabolizes arachidonic acid to 20-HETE, resulting in enhanced growth and spread of breast cancer cells^29, 30^. These studies suggest that CYP4Z1 could be a valuable marker to distinguish between benign and malignant breast and ovarian disease growths and could be a prognostic biomarker for malignant progression in these tissues. The data we present are consistent with previous reports and suggest that CYP4Z1 may be associated with the breast cancer health disparity among the three ethnicities.

#### CYP2A6

CYP2A6, the primary enzyme responsible for the oxidation of nicotine and cotinine, is also involved in the metabolism of several pharmaceuticals, carcinogens, and a number of coumarin-type alkaloids. Additional substrates metabolized by CYP2A6 include cyclophosphamide, ifosfamide, fluorouracil, and aflatoxin^24^. The mean expression values in RPM of CYP2A6 in adjacent normal tissue, all BRCA tissue, CA, AS, and AA breast tumor tissues are 5.63, 75.7, 96.3, 4.89, and 37.0, respectively. CYP2A6 expression is elevated in CA, AA tumor, and all BRCA tissue compared to normal breast tissue. CYP2A6 is significantly differentially expressed in all pair-wise comparisons by ethnicity (CA vs. AA; CA vs. AS; AA vs. AS), and in some stage and subtype specific comparisons (Table 1). The dysregulation of this gene may play a role in the health disparity between different ethnicities and in breast cancer in general.

#### CYP2A7

The differential expression pattern of this gene is similar to that of CYP2A6, wherein the mean normalized expression values in adjacent normal tissue, all BRCA tissue, CA, AS, and AA breast tumor tissues are 0.63, 30.8, 28.6, 1.80, and 24.9, respectively. The substrate of this enzyme is still unknown. CYP2A7 is part of a large cluster of CYP genes from the CYP2A subfamily located on chromosome 19q, and it is likely that differential expression of both CYP2A6 and CYP2A7 is caused by a single genetic variant.

#### CYP1B1

The enzyme encoded by CYP1B1 localizes to the endoplasmic reticulum (ER) and metabolizes procarcinogens, such as polycyclic aromatic hydrocarbons and 17beta-estradiol^31^. The mean expression values in adjacent normal tissue, all BRCA tissue, CA, AS, and AA breast tumor tissues are 87.0, 113, 126, 84.8, and 104, respectively. The differential expression pattern is similar to that of CYP2A6, but the scale of differential expression is much smaller for this gene. CYP1B1 expression is significantly higher in CA triple-negative BRCA (TNBC) than in AA TNBC (Table 1).

#### Alcohol dehydrogenase (ADH)

Several alcohol dehydrogenases are down-regulated in BRCA tissues compared to adjacent normal tissues, including ADH1A, ADH1B, ADH1C, and ADH4. Members of ADH enzyme family metabolize a wide variety of substrates, including ethanol, retinol, other aliphatic alcohols, hydroxysteroids, and lipid peroxidation products. It is unclear whether this differential expression plays any role in breast cancer or contributes to racial disparities in BRCA outcomes. The expression values for the down-regulated ADH genes are given in supplementary materials (Table S3).

#### UDP-glucuronosyltransferases (UGTs)

Glucuronidation, catalyzed by UGTs, is an important process of metabolism and detoxification of estrogens. Some UGTs have been reported to be differentially expressed in breast cancer^32^. Several UGT family genes are differentially expressed in various comparisons across the three race groups (Table S2).

### Drug and xenobiotics metabolism pathways

Pathway enrichment analyses in KEGG pathways using all the significantly differentially expressed DXME genes from AA, CA and AS BRCA samples were conducted for three different pair-wise combinations: AA vs. CA, AA vs. AS, and CA vs. AS. The analyses identified that the KEGG “pathway drug metabolism - cytochrome P450”, is significantly up-regulated in AA BRCA samples, compared to AS BRCA samples with an adjusted p-value of 0.0078. The pathway “Metabolism of xenobiotics by cytochrome P450” is also significantly up-regulated in AA as compared to AS BRCA samples with adjusted p-value of 0.026. Metabolic disorders of biological oxidation enzymes pathway (adjusted p-value = 0.0071) is significant between CA and AS BRCA tumor samples when also including Reactome pathways in the analysis. Differential analysis results for all the genes are used as input for Pathview analysis and the overview for drug metabolism pathway is given in Figures S1–S3 (Supplementary materials). Metabolism pathways for several anticancer drugs commonly used in the treatment of breast cancer (detailed below) were identified as being differentially regulated among CA, AA, and AS BRCA patients.

#### Tamoxifen metabolism

**Figure 2:**
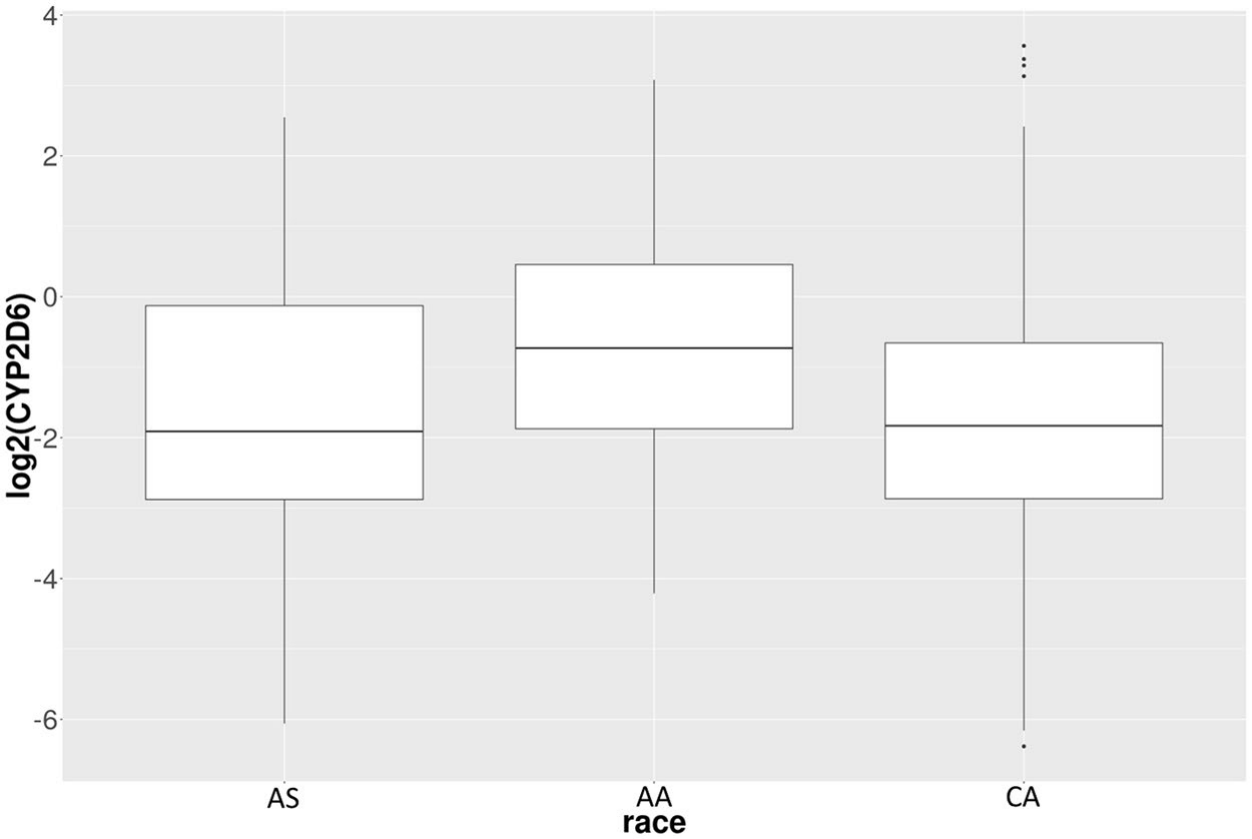
Expression of CYP2D6 in Asian American (AS), African American (AA), and Caucasian American (CA) breast cancer tissues. Y-axis is log2 of normalized expression values (in reads per million). The p-value for AA vs. AS comparison is 0.075, and the p-value for CA vs. AA comparison is 1.27e-06. The reason that the latter has much smaller p-value is because the sample sizes of AA and CA are much larger than that of AS.

**Figure 3:**
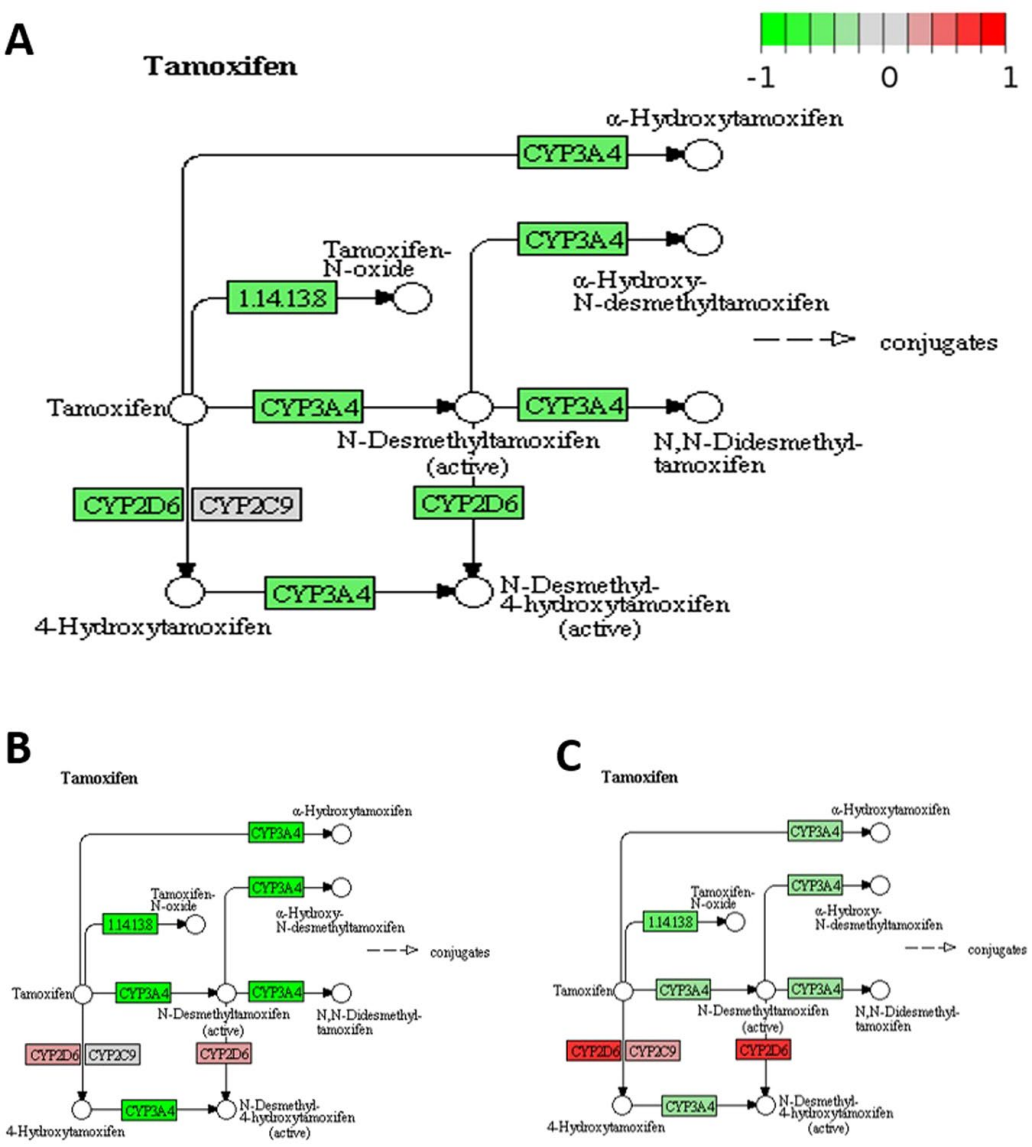
Differential gene expression in the tamoxifen metabolism pathway. (**A**) Asian American vs African American. Up or down regulation means increase or decrease of gene expression in Asian American BRCA patient tumor tissues. (**B**) Asian American vs Caucasian American. Up or down regulation means increase or decrease of gene expression in Asian American BRCA patient tumor tissues. (**C**) African American vs Caucasian American. Up or down regulation means increase or decrease of gene expression in African American BRCA patient tumor tissues. The pathway diagrams with differential gene expression were made using Pathview package^47^, where pathway diagrams were originally obtained from KEGG database^18–20^.

Tamoxifen is a selective estrogen receptor modulator which is used in the treatment and prevention of breast cancer, specifically ER-positive BRCA. Tamoxifen is metabolized in human body to the active metabolite 4-hydroxytamoxifen (4OHT) by several enzymes, including CYP2D6, CYD3A4, CYP1A1, CYP2B6, and CYP3A5^33^. Changes in expression of these genes may affect the concentration and bioavailability of active tamoxifen in breast tumors. It is known that patients with variant forms of the gene CYP2D6 may not receive full benefit from tamoxifen because of slower metabolism of the tamoxifen prodrug into active 4OHT^34, 35^. Furthermore, some patients, called “ultra-fast metabolizers”, metabolize tamoxifen too rapidly to keep enough active 4OHT available long enough to achieve the desired therapeutic effect. We report that expression of CYP2D6 is higher in AA breast tumor samples compared to AS and CA BRCA (Fig. 2). Other genes involved in tamoxifen metabolism are also differentially expressed in BRCA tissues in pair-wise comparisons among the three ethnicities (Table 1). For example, CYP3A4 and CYP2D6 are both down-regulated in AS BRCA compared to AA BRCA, indicating reduced activity of the tamoxifen metabolism pathway (Fig. 3).

#### Cyclophosphamide and Ifosfamide

Cyclophosphamide and ifosfamide are alkylating agents of the nitrogen mustard type that are commonly used as chemotherapy to treat various cancers, including breast cancer. As prodrugs, they are converted by CYP enzymes to form the metabolite 4-hydroxy cyclophosphamide that has chemotherapeutic activity. Multiple enzymes in the cyclophosphamide and ifosfamide metabolism pathways are differentially expressed in BRCA tissues among the three ethnic groups, including CYP2B6, CYP3A4, CYP3A5, and enzymes in classes 2.5.1.18, 1.1.1.1 and 1.2.1.5 (Figure S4). Similar to the tamoxifen metabolism pathway, AS BRCA had lower expression of genes in these pathways than either AA or CA BRCA.

#### Fluorouracil

Fluorouracil belongs to a family of drugs called antimetabolites and is widely used in the treatment of various cancers, including breast cancer. CYP2A6 metabolizes the prodrug tegafur into active fluorouracil (Figure S5). CYP2A6 expression is significantly lower in AS BRCA compared to AA and CA BRCA and is lower in AA BRCA compared to CA BRCA. In addition to CYP2A6, enzymes in several classes including class 3.5.4.5, class 2.4.2.4, class 2.4.2.3, class 2.7.1.48, and class 2.7.1.21 are also differentially expressed between CA, AA, and AS BRCA (Figure S5).

### Metabolism of xenobiotics by cytochrome P450 (CYP)

**Figure 4:**
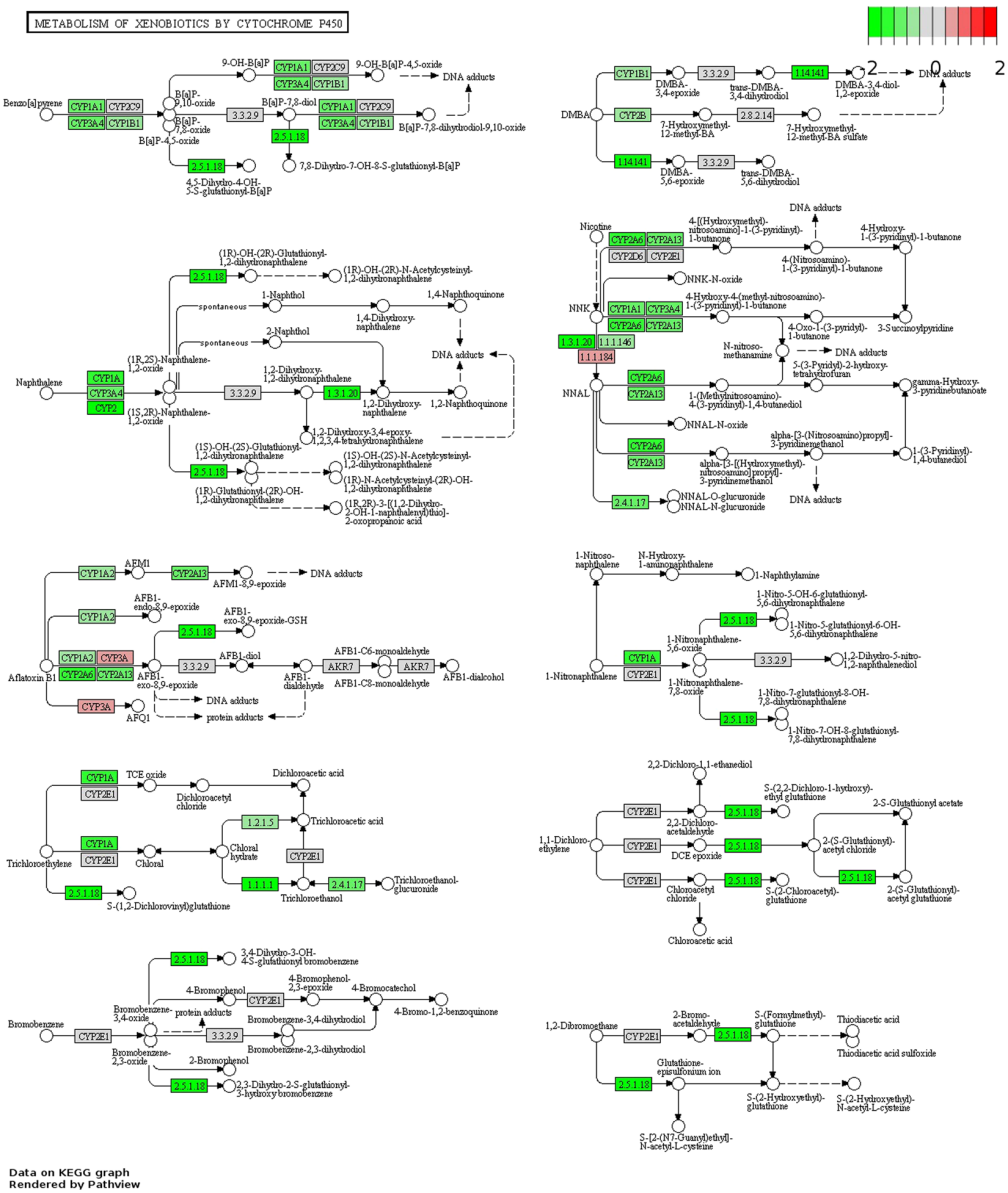
Differential gene expression in xenobiotics metabolism pathway between Asian American and Caucasian American. Up or down regulation means increase or decrease of gene expression in Asian American BRCA patient tumor tissues. The pathway diagram with differential gene expression was made using Pathview package^47^, where pathway diagrams were originally obtained from KEGG database^18–20^.

We next performed pathway analysis for pathways involved in xenobiotics metabolism by CYP genes. Metabolism of xenobiotics by CYP enzymes in breast tissues may play important roles in breast cancer risk. Several types of xenobiotics, including many carcinogens, are included in the KEGG pathways, including Benzo pyrene, DMBA, Naphthalene, Nicotine, Aflatoxin, 1-nitronaphthalene, trichloroethylene, 1,2-dichloroethylene, bromobenzene, and 1,2-dibromoethane. We found that AS breast tumor tissues have lower expression for the majority of the enzymes in xenobiotic metabolism pathways compared to both CA (Fig. 4) and AA (Figure S6, Supplementary material) breast tumor tissues. AA breast tumor has comparable number of differentially expressed genes in this pathway compared to CA breast tumor (Figure S7, Supplementary Material). Increased activities of these xenobiotics metabolism enzymes in CA or AA patients may produce extra amount of carcinogens in breast tissue and cause increased breast cancer risk. It is well known that AS have lower breast cancer incidence than CA and AA^1^, and the difference in the expression profiles of the xenobiotics metabolizing enzymes may play a role in that health disparity.

### SNPs associated with the differential expressions of DXME

An important question in disease studies is to identify the underlying genotypes directly responsible for an observed phenotype. In this study we focus on several phenotypes of interest: the outcomes disparity among CA, AA, and AS BRCA patients, ethnic disparity in BRCA risk, and expression profiles of DXME genes from breast cancer tissues. To identify genotypes associated with these phenotypes, we combined gene expression data with eQTL data from the GTEx project and genotype information from the 1000 Genomes project. Using this approach we searched for SNPs that may be associated with the differential expression of DXME genes in breast cancer tissues from CA, AA, and AS patients. To illustrate our approach we detail this analysis for identification of SNPs associated with differential expression of the CYP2D6 gene. We first identified 138 SNPs that associate with CYP2D6 expression in breast tissue from the GTEx eQTL database. In addition to SNP IDs we also obtained the effect size for each SNP. Using data from 1000 Genomes project we calculated the alternative allele frequency for each SNP in three different ethnic groups: African, Caucasian, and Asian populations. The allele frequencies and effect sizes are then used to calculate relative expression of each SNP (see Methods for details). In Table 2, we list the SNPs identified as associated with CYP2D6 expression (p-value ≤ 0.01). The SNPs were discovered using gene expression from AA and CA tumors and were independently confirmed in analysis of SNPs associated with CYP2D6 expression in AS BRCA (Table 2). These SNPs fall into two clusters of linkage disequilibrium with the first three in one cluster and the last three in another cluster. These SNPs are all close to one another and are distant from the CYP2D6 locus (chromosome 22, base location 42126499–42130906). Examination of the genomic region of these SNPs in the UCSC genome browser, revealed three genes in this region: SERHL, RRP7A, and SERHL2. The expression of these genes is not significantly correlated with that of CYP2D6, suggesting that regulation of CYP2D6 through this genomic region may not be related to the products of any of these three genes. Previous studies have mostly looked at variants on the coding regions or locations close to the coding region of DXME, which may affect the gene function by changing the protein sequence of the gene product. Our approach will likely find new SNPs that affect the function of CYP2D6 and other DXME genes by affecting trans-regulation of gene expression. SNPs such as these can be difficult to find using existing methods, and our approach illustrates an improved method for finding SNPs associated with gene expression by performing integrated secondary analyses on existing data. SNPs identified in this manner may be causal factors contributing to differential expression of DXME and may underlie ethnic disparities in BRCA incidence and outcomes.

**Table 2:** SNPs that may be associated with the differential expression of CYP2D6 in breast cancer cells.

SNP Id	Genomic location	Calculated diff. expression	Alt. allele freq. in AA	Alt. allele freq. in CA	Alt. allele freq. in AS	Effect size in eQTL	Distance to TSS
rs7245	22:42481849	0.2279	0.97	0.54	0.61	0.53	355 kb
rs5751220	22:42516205	0.2236	0.97	0.54	0.62	0.52	390 kb
rs6002626	22:42517989	0.2279	0.97	0.54	0.61	0.53	391 kb
rs714002	22: 42569024	0.2548	0.7	0.21	0.28	0.52	442 kb
rs713811	22:42569870	0.2496	0.69	0.21	0.27	0.52	443 kb
rs2011944	22:42569999	0.2444	0.68	0.21	0.27	0.52	443 kb

### Correlation between the expressions of some DXME genes with clinical variables

Using TCGA data analysis results obtained from the Broad Institute’s GDAC database we examined correlations between expression of CYP genes and clinical characteristics, including patient age, disease stage, node status, metastatic status, and survival time. We found that expressions of certain CYP genes is correlated with node status (N stage) of breast cancer including CYP2A6 (p-value = 0.025), CYP2C8 (p-value = 0.035), CYP2D6 (p-value = 0.027), CYP2J2 (p-value = 0.036), CYP3A7 (p-value = 0.026), and CYP4B1 (p-value = 0.046). Expression of CYP4Z1 is correlated with metastatic stage (M stage; p-value = 0.011). Expression of CYP4F12 correlates with T stage (p-value = 0.0029). Expression of CYP2E1 correlates with disease stage (p-value = 0.000742). The expression of several CYP genes correlates strongly with age, including CYP1B1 (p-value = 1.01E-08), CYP2A6 (p-value = 0.000198), CYP4X1 (p-value = 2.92E-08), and CYP4Z1 (p-value = 1.11E-09). Whether these correlations are associated with increased breast cancer risk as people get older needs to be investigated in future studies.

## Discussion

The involvement of DXME in cancer, particularly regarding contributions to the emergence of therapeutic resistance, is an area of active study. It has been reported that ovarian cancer cells can express functional taxane-metabolizing enzymes that enhance the ability of cancer cells to metabolize the chemotherapeutic agent docetaxel, representing a novel mechanism of chemotherapy resistance^36^. Additionally, it was also shown that human colorectal cancer cells are able to inactivate the anticancer drug paclitaxel through metabolism by CYP2C8 and CYP3A4, demonstrating an example of acquired therapeutic resistance through induction of DXME^37^. A study in lung tissue showed that DXME involved in the metabolism of anti-cancer drugs influence how tumors respond to chemotherapy, and suggested that DXME should be a factor to consider when determining therapeutic options^38^. In addition to their role in the metabolism of anticancer drugs, DXMEs, including cytochrome P450 genes (CYPs), may also have an important role in cancer initiation and progression. For example, high levels of CYP1B1 expression in breast tumor tissues was reported to be associated with significantly increased breast cancer risk^39^, based on the observation that high CYP1B1 expression in breast cancer cells can evoke changes in their response to drugs that are substrates of CYP1B1, thus influencing the metabolism or activation of environmental carcinogens^40^. Additionally, CYP2E1 expression was demonstrated to be a potential prognostic biomarker in breast cancer^40^.

It is well-established that rates of breast cancer incidence and mortality differ among different ethnic groups. Many studies have been conducted to understand the factors underlying this phenomenon, and it has been suggested that genetic factors may play important roles in this health disparity^7–10^.

In this study, we performed an integrative analysis of gene expression, eQTL, SNP, and pathway data associated with the expression of drug and xenobiotics metabolizing enzymes (DXME) in breast cancer (BRCA) tissues from patients across three major ethnic groups, Caucasian American (CA), African American (AA), and Asian American (AS). These analyses (1) revealed that expression of DXME genes in breast cancer tissue differs among CA, AA, and AS patients; (2) indicated that pathways involving DXME genes are differentially regulated in CA, AA, and AS breast cancer patients; and (3) identified SNPs that may not only be associated with the differentially expressed DXME genes and pathways, but that also may contribute to BRCA health disparities among these three ethnic groups. These data provide further evidence clarifying the biological and genetic contributions to observed breast cancer disparities among patients from different ethnic groups.

We report that 42 DXME genes are significantly differentially expressed in at least one group-wise comparison among CA, AA, and AS breast cancers. Moreover, DXME expression from AS BRCA was the most significantly different compared to CA or AA BRCA. We also found that AS breast tumors tend to exhibit gene expression indicative of repression of xenobiotic metabolism pathways compared to both AA and CA breast tumors.

In particular, the CYP genes may critically contribute to breast cancer health disparities between CA, AA, and AS populations. Using CYP2D6 as an example, we identified several SNPs that may be associated with the differential expression of CYP2D6 among CA, AA, and AS breast cancer tissues. This approach could be applied to other genes to identify more expression-associated SNPs in breast cancer. As many associations indicate trans-regulation, these associations may help better understand the gene and protein interactions and driver genes in breast cancer. In addition to contributing to health disparities, these SNPs may also be associated with breast cancer risk and progression in general. The specific role of these SNPs in regulation of DXME expression and pathway activation needs to be elucidated in future studies.

A previous study examined the expression profile of 21 CYP genes in breast cancer cells using a tissue microarray containing 170 breast cancers and reported that the highest percentage of strong immunopositivity was seen for CYP4X1, CYP2S1 and CYP2U1, while CYP2J and CYP3A43 frequently displayed no immunoreactivity^17^. In this study we report consistent observations regarding CYP4X1 and CYP3A43, although expression patterns for other CYP genes were inconsistent with the previous report. This discrepancy may be due to different experimental method (IHC) that was used to measure CYP protein expression in the previous study^17^. We report that the CYP genes with highest expression, ranked by the mean expression in tumor tissues for all patients, are: CYP1B1 (mean normalized expression 112.8), CYP4Z1 (92.2), CYP2A6 (75.7), CYP4X1 (65.1), CYP4B1 (31.5), CYP2A7 (30.8), and CYP4F8 (11.6). The CYP genes with the lowest expression in the TCGA breast cancer cohort were: CYP1A2 (0.010), CYP3A43 (0.029), CYP3A7 (0.033), CYP2C9 (0.044) and CYP3A4 (0.044).

In this study, we took an integrative approach using multiple types of data. The ethnic information available through public data repositories, including the TCGA, serves as a key link through which we were able to: (1) identify patterns of DXME expression within breast tumor tissues for a diverse breast cancer patient population; (2) perform differential gene expression analysis to identify genes that may be associated with breast cancer health disparities and breast cancer incidence and progression in general; and (3) combine the data from the 1000 Genomes project to identify SNPs associated with DXME expression/pathway activation and ethnic disparities in breast cancer. This novel integrative approach can be applied to other cancer types to discover genetic features that underlie disparities in disease incidence and outcomes.

Precision cancer medicine aims to provide personalized cancer therapies to individuals according to their characteristic responses to available therapies. It requires in-depth understanding of cancer heterogeneity, which manifests itself at both phenotype (response to treatment) and genotype (including both genetics and epigenetics) levels. While the goal is usually to understand how genotype variations cause the phenotype differences, understanding the heterogeneity at gene expression levels can provide very valuable links between heterogeneities at phenotype and genotype (or epigenomic) levels. Genome wide association studies (GWAS) examining large number of genomic variants often have to deal with multiple testing issues, which tend to produce large numbers of false positives. Race, as a natural stratification of human population, provides a different angle to investigate the heterogeneity at phenotypic, gene expression, and genotypic levels when race information is available across data sets at these different levels. The differentially expressed genes among different race groups discovered in this study are likely relevant in precision medicine. Some can be potential candidates for biomarkers for personalized cancer therapy. If clinically validated, they may be used independent of race information of the patients.

Better mechanistic understanding of how differences in DXME expression and pathway regulation contribute to BRCA incidence and outcome disparities will facilitate the development of better personalized chemoprevention strategies. Our study suggests that development of personalized treatment strategies and prognostic/diagnostic tools incorporating DXME should take multiple genetic factors into account simultaneously and should consider both germline genotypes and tumor-specific mutations and expression profiles

## Methods

### RNA-Seq Data from TCGA

Data used in this study were downloaded from Insilicom’s BioKDE platform (insilicom.com), where processed de-identified genomic and clinical data were downloaded from TCGA (the Cancer Genome Atlas) and integrated into a relational database for convenient queries. The de-identified patient data included up to 20483 RNA sequence-derived gene expression values and clinical characteristics (*e*.*g*., age, cancer stage, receptor status). In total, there are 728 Caucasian American, 143 African American, and 57 Asian American breast tumor tissue samples, as well as 103, 6, and 1 normal breast tissue samples from the respective ethnic groups. All the pair-wise comparisons were first matched by age and tumor stage. For AA vs. AS comparison, 53 AS samples were matched with 103 AA samples; for CA vs. AS comparison, 57 AS samples were matched with 399 CA samples; and for CA vs. AA comparison, 138 AA samples were matched with 552 CA samples. Subtypes were assigned to patient samples according to established methods (27). Normalized patient gene expression counts in reads per million (RPM) were used unless otherwise noted.

### Differential Gene Expression

There are many methods for differential gene expression analysis based on different distribution assumptions^41–44^. We chose to use DESeq2, an R Bioconductor package, for our analyses^45, 46^. Patient gene counts input into DESeq2 were rounded to the nearest whole number but not normalized. Jobs were run on the Insilicom BioKDE server (insilicom.com). Expression values are reported as Reads Per Million (RPM). For AS vs. CA comparisons, changes in expression are reported as relative to the CA group. For AS vs. AA comparisons, changes in expression are reported as relative to the AA group. For AA vs. CA comparisons, changes in expression are reported as relative to the CA group.

### Sample Matching

All the comparisons done in this study were matched by age and stage using the R package Matching. The matching was done differently from an earlier study^10^. Stage was matched exactly and age was matched in a way that any patients are considered matched if the difference of their ages is less than or equal to 10. This is more flexible than the three categories used in a previous study. The R Matching package also performs optimal matching once the ratio of the two types of subject are determined. To find the optimal ratio, we maximize the quantity, *nm*/(*n* + *m*), where *n* is the number of patients of one ethnicity and *m* is the number of patients of the other ethnicity. In this setup, we assume the power calculation of testing differential gene expression can be approximated by two sample test of two normal samples with different sample sizes. We found that the new matching method produced notable differences, but the exact effect of it was not evaluated systematically.

### Pathway visualization

The visualization of differentially regulated pathways was performed using R’s Pathview package^47^.

### Association of SNPs to gene expression in breast cancer

Combining data obtained from GTEx (Genotype-Tissue Expression) eQTL (Expression Quantitative Trait Loci) studies and 1000 Genomes project with the gene expression data from TCGA, we inferred potential associations of certain SNPs to gene expressions in breast cancer. Firstly, given a gene of interest, SNPs that are associated with expression of this gene are obtained from GTEx data portal for eQTL (http://www.gtexportal.org/home/eqtl) with breast tissue as the condition. Secondly, the SNPs are used to compute the allele frequencies for ethnicities of interest using data from 1000 Genomes project (http://www.1000genomes.org/). Next, the effect sizes from eQTL and the allele frequencies are used to compute relative expressions of the gene (marginal expression without conditioning on other factors). Finally, the relative expressions computed from allele frequencies and eQTL effect sizes are tested against the actual expressions observed in TCGA data.

We use CYP2D6 as an example to illustrate how we can combine gene expression data with eQTL data and allele frequency data to identify potentially associated SNPs with gene expression in breast cancer cells. Given that CYP2D6 is over-expressed in AA BRCA tumor tissues compared to CA and AS tissues, only those SNPs that support this observation can be associated with this differential expression. Although we could use data from all three ethnic groups to perform a more sophisticated analysis, we use only AA and CA data to discover candidate SNPs and use AS data for validation purpose. We first found all the SNPs that are associated with expression of CYP2D6 in breast tissues from eQTL data in GTEx project. We then compute the alternative allele frequencies from 1000 Genomes project for African, European, and Asian populations. The relative frequency was used to compute relative differential expression in terms of effect size using the effect size information from eQTL data. Those effect sizes that have p-values smaller than 0.01 were saved for further analysis.

The two sample t-test statistics is calculated by $t=\frac{{X}_{1}-{X}_{2}}{{s}_{{X}_{1}{X}_{2}}\sqrt{\frac{1}{{n}_{1}}+\frac{1}{{n}_{2}}}}$, where X_1_ is the mean of one sample, X_2_ is the mean of the second sample, s_X1X2_ are their sample standard deviation, n_1_ is the size of sample one, and n_2_ is the size of sample two. The calculated effect size (calculated differential expression) is obtained by the effect size in eQTL multiplied by the difference in allele frequency of two populations. This calculated effect size is used to approximate the $\frac{{X}_{1}-{X}_{2}}{{s}_{{X}_{1}{X}_{2}}}$ in the t-statistics. n_1_ and n_2_ are obtained from the gene expression data. For CA the sample size is 552 and for AA the sample size is 138. If we want to have p-values ≤ 0.01, then the calculated effect size need to be at least greater than 0.22. We considered all the SNPs with calculated effect size ≥0.22 for further analysis.

### False Discovery Rates (FDR)

Adjusted p-value ≤ 0.05 and fold change ≥2 were used for reporting significantly differentially expressed genes (unless otherwise noted) to reduce the number of false positives. Where possible, the Benjamini-Hochberg procedure was used to control for FDR and is reported in the results^48^. Benjamini-Hochberg is used in DESeq2 output by default. P-values that have been adjusted are referred to as adjusted P-values or p_adj.

The study was approved by the Florida State University Research Ethics Committee. All data was drawn from either The Cancer Genome Atlas (TCGA) or The 1000 Genomes Project, each of which follow the appropriate guidelines. We have received permission to use data from TCGA; data from The 1000 Genomes Project is free to use under the Fort Lauderdale agreement.

## Electronic supplementary material

Supplementary Material 1

DXME gene expression summary

Differentially expressed genes

## References

[1] American Cancer Society Cancer Facts & Figures 2016 (American Cancer Society, Atlanta, 2016).

[2] Schootman M (2010). Temporal trends in geographic disparities in small-area breast cancer incidence and mortality, 1988 to 2005. Cancer Epidemiol Biomarkers Prev.

[3] Gerend MA, Pai M (2008). Social determinants of Black-White disparities in breast cancer mortality: a review. Cancer Epidemiol Biomarkers Prev.

[4] Harper S (2009). Trends in area-socioeconomic and race-ethnic disparities in breast cancer incidence, stage at diagnosis, screening, mortality, and survival among women ages 50 years and over (1987–2005). Cancer Epidemiol Biomarkers Prev.

[5] Echeverria SE, Borrell LN, Brown D, Rhoads G (2009). A local area analysis of racial, ethnic, and neighborhood disparities in breast cancer staging. Cancer Epidemiol Biomarkers Prev.

[6] Iqbal J, Ginsburg O, Rochon PA, Sun P, Narod SA (2015). Differences in breast cancer stage at diagnosis and cancer-specific survival by race and ethnicity in the United States. JAMA.

[7] Martin DN (2009). Differences in the tumor microenvironment between African-American and European-American breast cancer patients. PloS one.

[8] Field LA (2012). Identification of differentially expressed genes in breast tumors from African American compared with Caucasian women. Cancer.

[9] Grunda JM (2012). Differential expression of breast cancer-associated genes between stage- and age-matched tumor specimens from African- and Caucasian-American Women diagnosed with breast cancer. BMC Res Notes.

[10] Paul AS, Jennifer L, Mark DR, Qing-Xiang AS, Jinfeng Z (2013). Differentially expressed transcripts and dysregulated pathways in African American Breast Cancer. PLoS One.

[11] Shi Y (2017). Integrative Comparison of mRNA Expression Patterns in Breast Cancers from Caucasian and Asian Americans with Implications for Precision Medicine. Cancer Research.

[12] Zhou F (2011). Toward a new age of cellular pharmacokinetics in drug discovery. Drug Metab Rev.

[13] Rochat B (2005). Role of cytochrome P450 activity in the fate of anticancer agents and in drug resistance: focus on tamoxifen, paclitaxel and imatinib metabolism. Clin Pharmacokinet.

[14] Rochat B (2009). Importance of influx and efflux systems and xenobiotic metabolizing enzymes in intratumoral disposition of anticancer agents. Curr Cancer Drug Targets.

[15] Michael M, Doherty MM (2007). Drug metabolism by tumours: its nature, relevance and therapeutic implications. Expert Opin Drug Metab Toxicol.

[16] Yasuda SU, Zhang L, Huang SM (2008). The role of ethnicity in variability in response to drugs: focus on clinical pharmacology studies. Clin Pharmacol Ther.

[17] Murray GI, Patimalla S, Stewart KN, Miller ID, Heys SD (2010). Profiling the expression of cytochrome P450 in breast cancer. Histopathology.

[18] Kanehisa M, Sato Y, Kawashima M, Furumichi M, Tanabe M (2016). KEGG as a reference resource for gene and protein annotation. Nucleic Acids Res.

[19] Kanehisa M, Furumichi M, Tanabe M, Sato Y, Morishima K (2017). KEGG: new perspectives on genomes, pathways, diseases and drugs. Nucleic Acids Res.

[20] Kanehisa M, Goto S (2000). KEGG: kyoto encyclopedia of genes and genomes. Nucleic Acids Res.

[21] Zhou SF, Liu JP, Chowbay B (2009). Polymorphism of human cytochrome P450 enzymes and its clinical impact. Drug Metab Rev.

[22] Preissner SC (2013). Polymorphic cytochrome P450 enzymes (CYPs) and their role in personalized therapy. PLoS One.

[23] Rodriguez-Antona C, Gomez A, Karlgren M, Sim SC, Ingelman-Sundberg M (2010). Molecular genetics and epigenetics of the cytochrome P450 gene family and its relevance for cancer risk and treatment. Hum Genet.

[24] Raunio H, Rautio A, Gullsten H, Pelkonen O (2001). Polymorphisms of CYP2A6 and its practical consequences. Br J Clin Pharmacol.

[25] Higgins MJ, Stearns V (2010). CYP2D6 polymorphisms and tamoxifen metabolism: clinical relevance. Curr Oncol Rep.

[26] Johnson N (2012). CYP3A variation, premenopausal estrone levels, and breast cancer risk. J Natl Cancer Inst.

[27] Rieger MA (2004). Identification of a novel mammary-restricted cytochrome P450, CYP4Z1, with overexpression in breast carcinoma. Cancer Res.

[28] Savas U, Hsu MH, Griffin KJ, Bell DR, Johnson EF (2005). Conditional regulation of the human CYP4X1 and CYP4Z1 genes. Arch Biochem Biophys.

[29] Yu W (2012). Increased expression of CYP4Z1 promotes tumor angiogenesis and growth in human breast cancer. Toxicol Appl Pharmacol.

[30] Zheng L, Li X, Gu Y, Lv X, Xi T (2015). The 3′UTR of the pseudogene CYP4Z2P promotes tumor angiogenesis in breast cancer by acting as a ceRNA for CYP4Z1. Breast Cancer Res Treat.

[31] Tang YM (1996). Isolation and characterization of the human cytochrome P450 CYP1B1 gene. J Biol Chem.

[32] Starlard-Davenport A, Lyn-Cook B, Radominska-Pandya A (2008). Novel identification of UDP-glucuronosyltransferase 1A10 as an estrogen-regulated target gene. Steroids.

[33] Cronin-Fenton DP, Damkier P, Lash TL (2014). Metabolism and transport of tamoxifen in relation to its effectiveness: new perspectives on an ongoing controversy. Future Oncol.

[34] Goetz MP (2005). Pharmacogenetics of tamoxifen biotransformation is associated with clinical outcomes of efficacy and hot flashes. J Clin Oncol.

[35] Beverage JN, Sissung TM, Sion AM, Danesi R, Figg WD (2007). CYP2D6 polymorphisms and the impact on tamoxifen therapy. J Pharm Sci.

[36] DeLoia JA (2008). Expression and activity of taxane-metabolizing enzymes in ovarian tumors. Gynecol Oncol.

[37] Garcia-Martin E (2006). Acquired resistance to the anticancer drug paclitaxel is associated with induction of cytochrome P450 2C8. Pharmacogenomics.

[38] Leclerc J (2011). Xenobiotic metabolism and disposition in human lung: transcript profiling in non-tumoral and tumoral tissues. Biochimie.

[39] Wen W (2007). Expression of cytochrome P450 1B1 and catechol-O-methyltransferase in breast tissue and their associations with breast cancer risk. Cancer Epidemiol Biomarkers Prev.

[40] Vaclavikova R (2007). RNA expression of cytochrome P450 in breast cancer patients. Anticancer Res.

[41] Marioni JC, Mason CE, Mane SM, Stephens M, Gilad Y (2008). RNA-seq: an assessment of technical reproducibility and comparison with gene expression arrays. Genome research.

[42] Wang L, Feng Z, Wang X, Zhang X (2010). DEGseq: an R package for identifying differentially expressed genes from RNA-seq data. Bioinformatics.

[43] Nagalakshmi U (2008). The transcriptional landscape of the yeast genome defined by RNA sequencing. Science.

[44] Robinson MD, Smyth GK (2007). Moderated statistical tests for assessing differences in tag abundance. Bioinformatics.

[45] Anders S, Huber W (2010). Differential expression analysis for sequence count data. Genome biology.

[46] Gentleman RC (2004). Bioconductor: open software development for computational biology and bioinformatics. Genome biology.

[47] Luo W, Brouwer C (2013). Pathview: an R/Bioconductor package for pathway-based data integration and visualization. Bioinformatics.

[48] Benjamini Y, Hochberg Y (1995). Controlling the False Discovery Rate - a Practical and Powerful Approach to Multiple Testing. J Roy Stat Soc B Met.

