# Supplementary material for: Tumoral expression of drug and xenobiotic metabolizing enzymes in breast cancer patients of different ethnicities with implications to personalized medicine
Source: Sci Rep. 2017 Jul 6;7:4747. doi: 10.1038/s41598-017-04250-2 (PMC5500564; doi:10.1038/s41598-017-04250-2)

**Tumoral expression of drug and xenobiotic metabolizing enzymes in breast cancer patients of different ethnicities with implications to personalized medicine**

**Yan Li, Albert Steppi, Yidong Zhou, Feng Mao, Philip Craig Miller, Max M He, Tingting Zhao, Qiang Sun, Jinfeng Zhang**

**Supplementary Materials**

**Breast Cancer Subtypes**

In clinical practice, BRCA is divided into subgroups based on the expression of the estrogen receptor (ER), progesterone receptor (PR), and the status of gene amplification of human epidermal growth factor 2 receptor (HER2). The four predominant subtypes reported in the literature are categorized based on these receptors: luminal A (ER+ and/or PR+, HER2-), luminal B (ER+ and/or PR+, HER2+), HER2 (ER-, PR-, HER2+), and triple negative (ER-, PR-, HER2-). A subtype of triple negative breast cancer (TNBC) is the basal or basal-like phenotype that is negative for all three receptors but expresses specific basal markers (cytokeratin 5/6, cytokeratin 14, cytokeratin 17, and epidermal growth factor receptor) . This phenotype is more likely to undergo metastasis and is associated with poorer prognosis . Recent large-scale genomic studies have suggested that the whole spectrum of breast cancer subtypes extends beyond the four commonly known subtypes .

**Methods**

Differential Gene Expression: There are many methods for differential gene expression analysis based on different distribution assumptions . We chose to use DESeq2, an R Bioconductor package, for our analyses . Patient gene counts input into DESeq2 were rounded to the nearest whole number but not normalized. Jobs were run on the Insilicom BioKDE server (insilicom.com). For AS vs CA comparison, increased (or decreased) expression means the expression of AS patients are higher (or lower) than the expression of CA patients; For AS vs AA comparison, increased (or decreased) expression means the expression of AS patients are higher (or lower) than the expression of AA patients; For CA vs AA comparison, increased (or decreased) expression means the expression of AA patients are higher (or lower) than the expression of CA patients.

Sample Matching: All the comparisons done in this study were matched by age and stage. R package Matching was used for this purpose. The matching was done differently from the earlier study . The stage was matched exactly and age was matched in a way that any patients are considered matched if the difference of their ages is smaller or equal to 10. This is more flexible than the three categories used in the earlier study. Matching package also perform optimal matching once the ratio of the two types of subject are determined. To find the optimal ratio, we maximize the quantity, *nm*/(*n*+*m*), where *n* is the number of patients of one race and *m* is the number of patients of the other race. In this setup, we assume the power calculation of testing differential gene expression can be approximated by two sample test of two normal samples with different sample sizes. We found that the new matching method produced notable differences, but the exact effect of it was not evaluated systematically.

Pathway visualization: The visualization of differentially regulated pathways were made possible using R’s Pathview package. It allows users to easily spot up or down regulated genes in a pathway to understand how it is differentially regulated.

Association of SNPs to gene expression in breast cancer: Combining data obtained from GTEx (Genotype-Tissue Expression) eQTL (Expression Quantitative Trait Loci) studies and 1000 Genomes project with the gene expression data from TCGA, we infer potential associations of certain SNPs to gene expressions in breast cancer. Firstly, given a gene of interest, SNPs that are associated with expression of this gene are obtained from GTEx data portal for eQTL (<http://www.gtexportal.org/home/eqtl>) with breast tissue as the condition; secondly, the SNPs are used to compute the allele frequencies for races of interest using data from 1000 Genomes project (<http://www.1000genomes.org/>); thirdly, the effect sizes from eQTL and the allele frequencies are used to compute relative expressions of the gene (marginal expression without conditioning on other factors); finally, these relative expressions computed from allele frequencies and eQTL effect sizes are tested against the actual expressions observed in TCGA data.

We use CYP2D6 as an example to illustrate how we can combine gene expression data with eQTL data and allele frequency data to identify potentially associated SNPs with gene expression in breast cancer cells. Given that CYP2D6 is up-regulated in AA BRCA tumor tissues compared to CA and AS tissues only those SNPs that support such observation can be associated with this differential expression. Although we could use data from all three races to perform a more sophisticated analysis, we use only AA and CA data to discover candidate SNPs and use AS data for validation purpose. We first found all the SNPs that are associated with expression of CYP2D6 in breast tissues from eQTL data in GTEx project. We then compute the alternative allele frequencies from 1000 Genomes project for African, European, and Asian populations. The relative frequency was used to compute relative differential expression in terms of effect size using the effect size information from eQTL data. Those effect sizes that have p-values smaller than 0.01 were saved for further analysis.

The two sample t-test statistics is calculated by
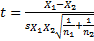
 , where X1 is the mean of one sample, X2 is the mean of the second sample, s_X1X2_ are their sample standard deviation, n1 is the size of sample one, and n2 is the size of sample two. The calculated effect size (calculated differential expression) is obtained by the effect size in eQTL multiplied by the difference in allele frequency of two populations. This calculated effect size is used to approximate the
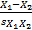
 in the t-statistics. n1 and n2 are obtained from the gene expression data. For CA the sample size is 552 and for AA the sample size is 138. If we want to have p-values <= 0.01, then the calculated effect size need to be at least greater than 0.22. We keep all the SNPs with calculated effect size >= 0.22 for further analysis.

False Discovery Rates (FDR): Adjusted p-value <= 0.05 and fold change >= 2 were used for reporting significantly differentially expressed genes (unless otherwise noted) to reduce the number of false positives. If possible, the Benjamini-Hochberg procedure was used to control for FDR and is reported in the results . Benjamini-Hochberg is used in DESeq2 output by default. P-values that have been adjusted are referred to as adjusted P-values or P_adj_.


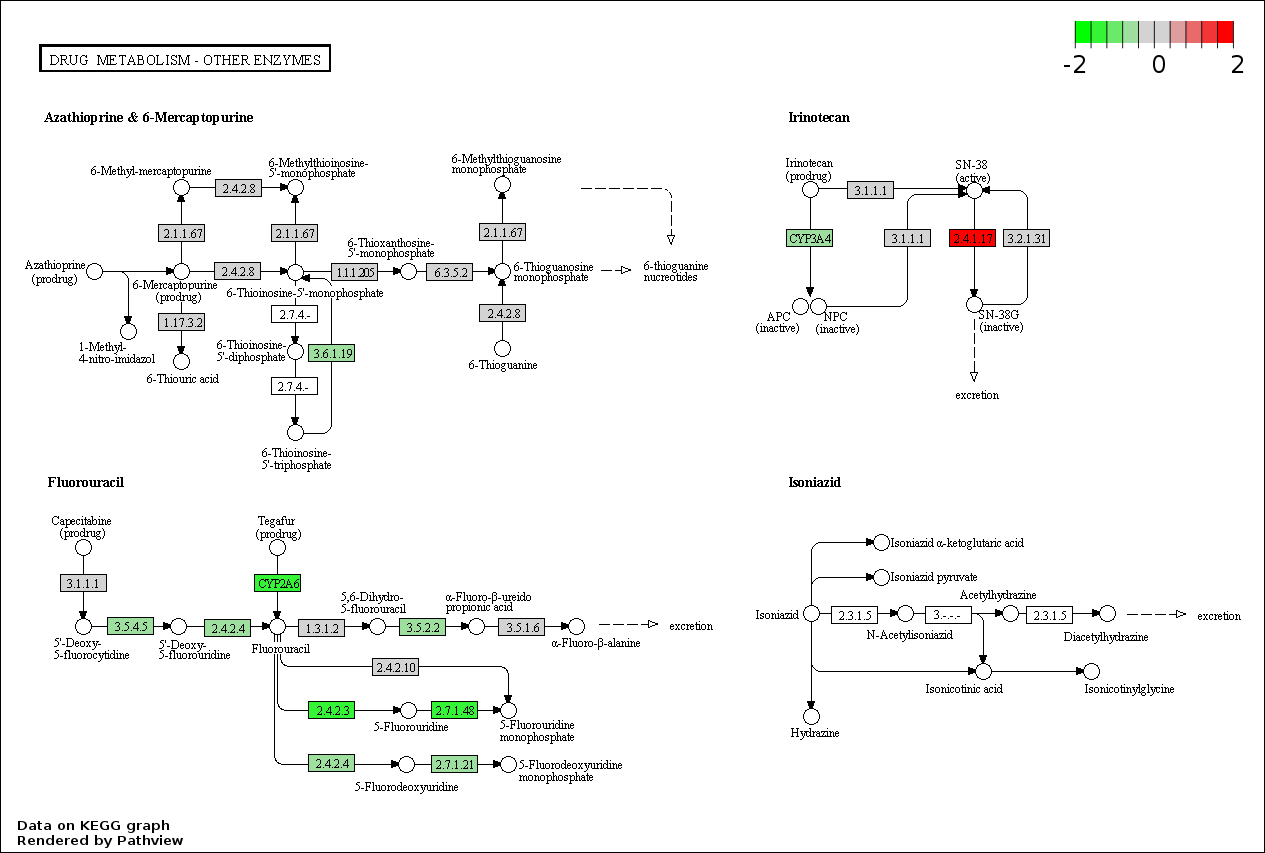


Figure S1. Pathview diagram for Drug metabolism - cytochrome P450 pathway. Comparison between AA and AS. The pathway diagram with differential gene expression was made using Pathview package[^43^](#_ENREF_43), where pathway diagrams were originally obtained at KEGG database[^45-47^](#_ENREF_45).


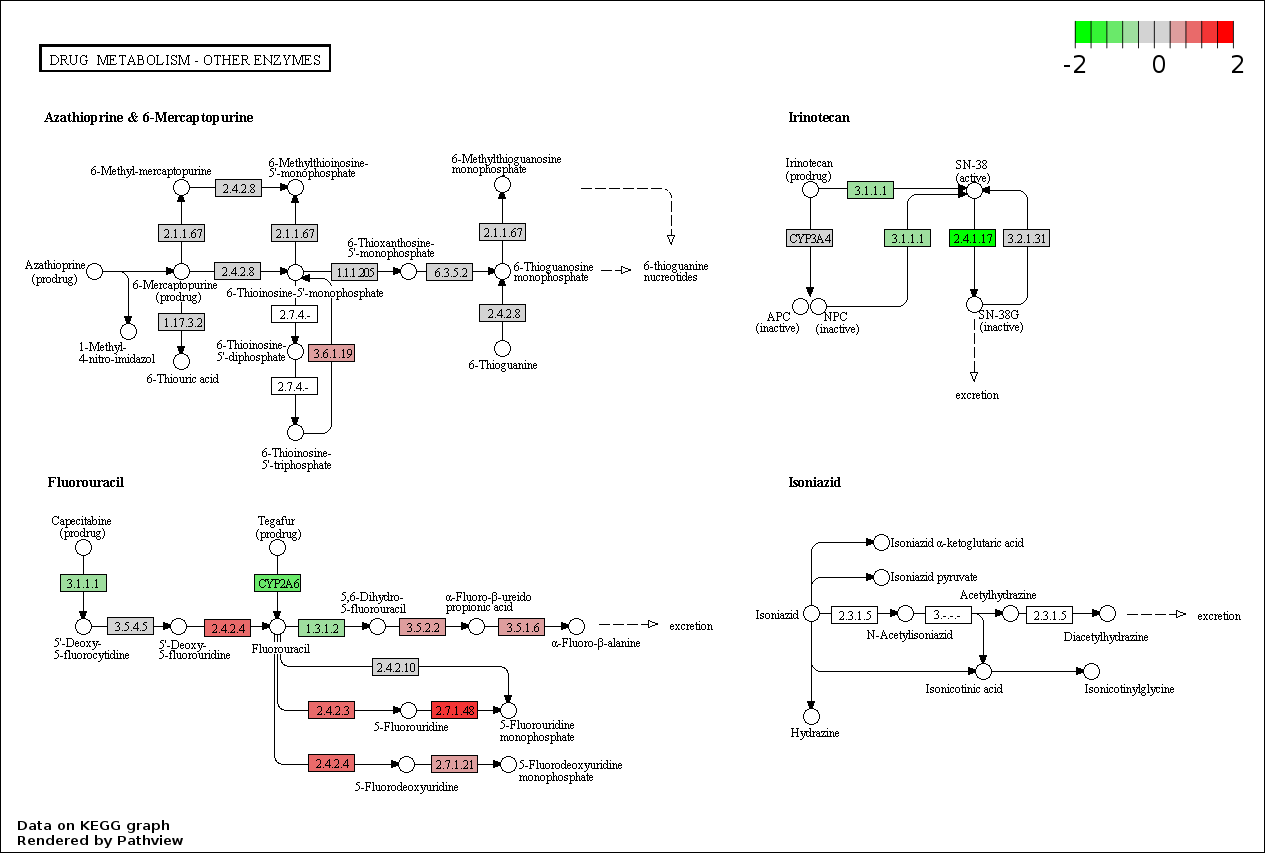


Figure S2. Pathview diagram for Drug metabolism - cytochrome P450 pathway. Comparison between AA and CA. The pathway diagram with differential gene expression was made using Pathview package[^43^](#_ENREF_43), where pathway diagrams were originally obtained at KEGG database[^45-47^](#_ENREF_45).


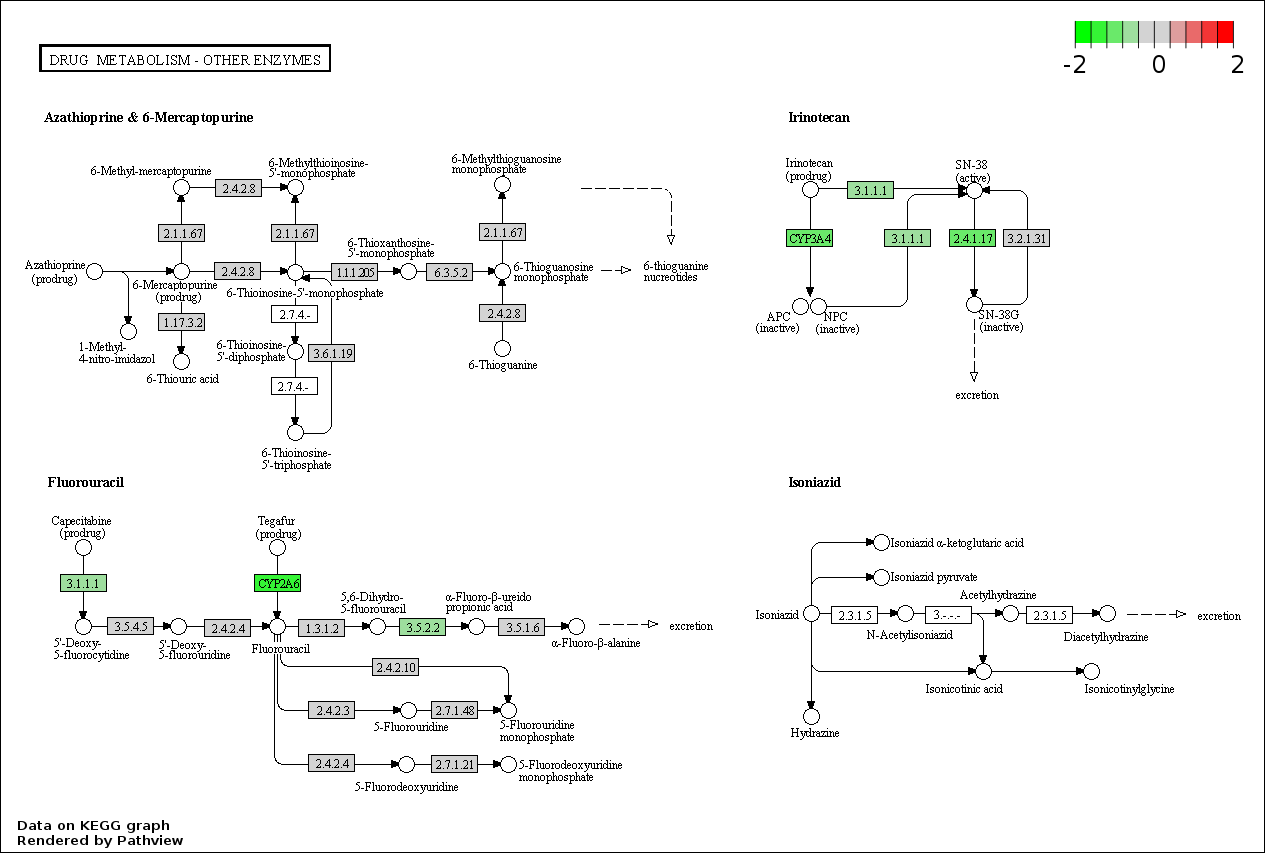


Figure S3. Pathview diagram for Drug metabolism - cytochrome P450 pathway. Comparison between CA and AS. The pathway diagram with differential gene expression was made using Pathview package[^43^](#_ENREF_43), where pathway diagrams were originally obtained at KEGG database[^45-47^](#_ENREF_45).


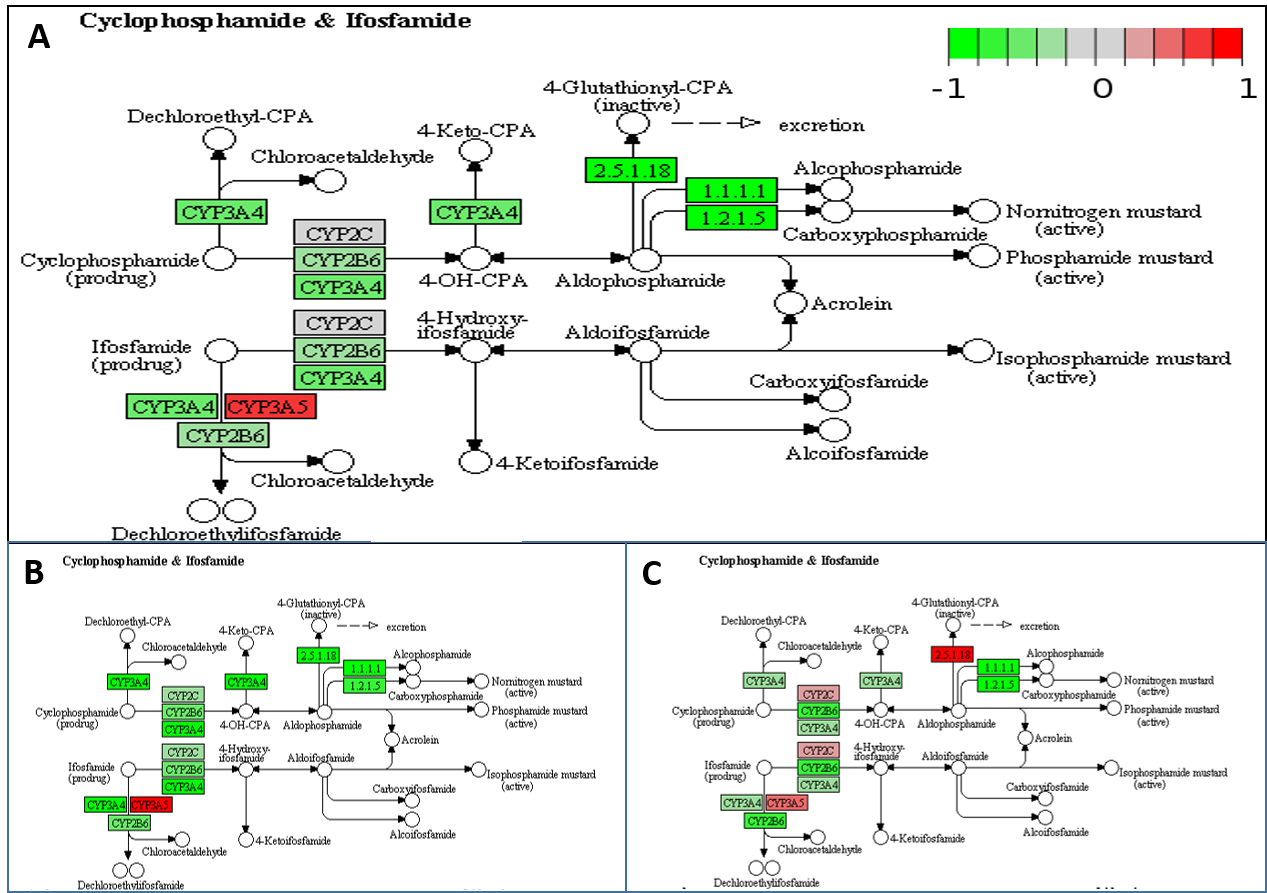


Figure S4. Differential gene expression in cyclophosphamide and ifosfamide metabolism pathway. (A) Asian American vs African American. Up or down regulation means increase or decrease of gene expression in Asian American BRCA patient tumor tissues. (B) Asian American vs Caucasian American. Up or down regulation means increase or decrease of gene expression in Asian American BRCA patient tumor tissues. (C) African American vs Caucasian American. Up or down regulation means increase or decrease of gene expression in African American BRCA patient tumor tissues. The pathway diagram with differential gene expression was made using Pathview package[^43^](#_ENREF_43), where pathway diagrams were originally obtained at KEGG database[^45-47^](#_ENREF_45).


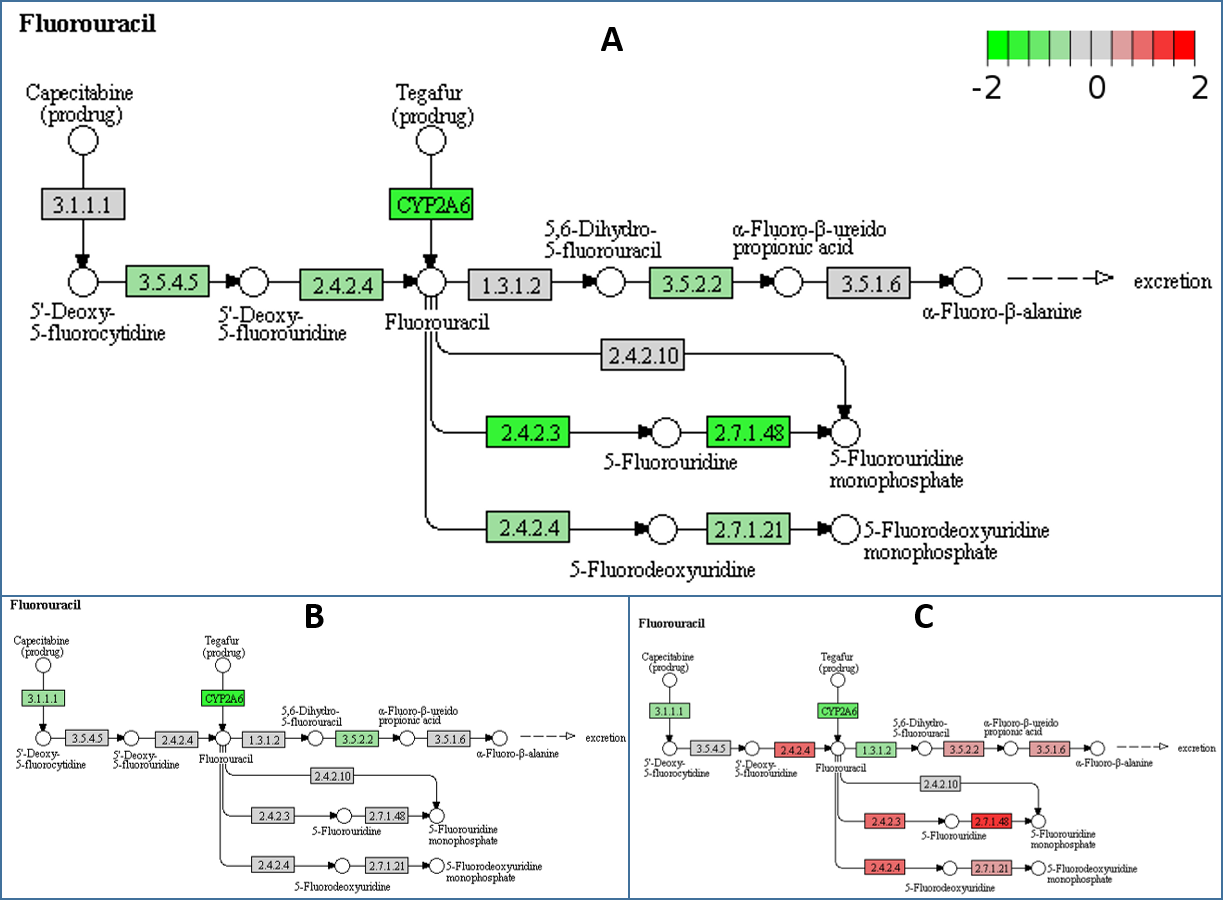


Figure S5. Differential gene expression in Fluorouracil metabolism pathway. (A) Asian American vs African American. Up or down regulation means increase or decrease of gene expression in Asian American BRCA patient tumor tissues. (B) Asian American vs Caucasian American. Up or down regulation means increase or decrease of gene expression in Asian American BRCA patient tumor tissues. (C) African American vs Caucasian American. Up or down regulation means increase or decrease of gene expression in African American BRCA patient tumor tissues. The pathway diagram with differential gene expression was made using Pathview package[^43^](#_ENREF_43), where pathway diagrams were originally obtained at KEGG database[^45-47^](#_ENREF_45).


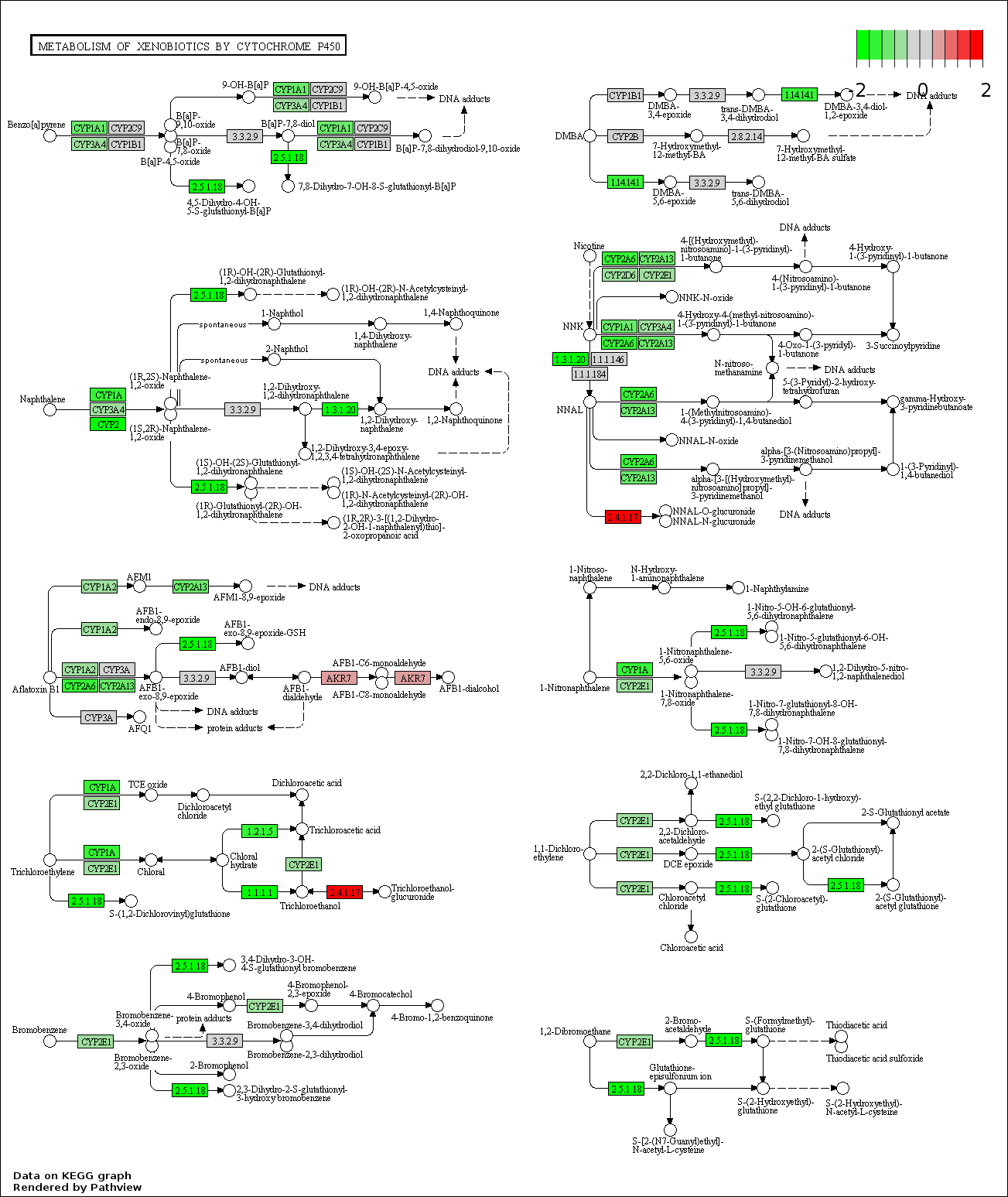


Figure S6. Differential gene expression in xenobiotics metabolism pathway between Asian American and African American. Up or down regulation means increase or decrease of gene expression in Asian American BRCA patient tumor tissues. The pathway diagram with differential gene expression was made using Pathview package[^43^](#_ENREF_43), where pathway diagrams were originally obtained at KEGG database[^45-47^](#_ENREF_45).


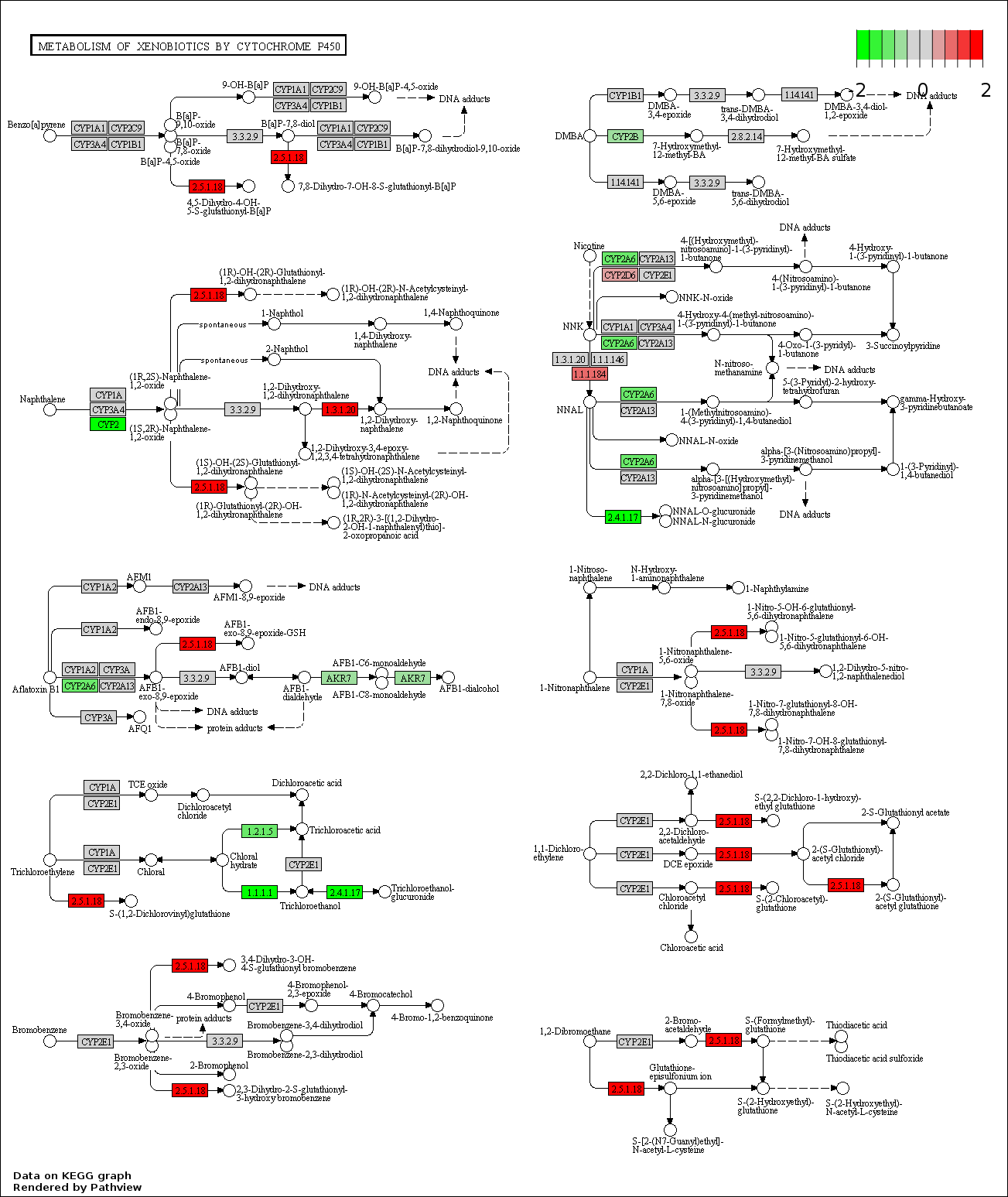


Figure S7. Differential gene expression in xenobiotics metabolism pathway between African American and Caucasian American. Up or down regulation means increase or decrease of gene expression in African American BRCA patient tumor tissues. The pathway diagram with differential gene expression was made using Pathview package[^43^](#_ENREF_43), where pathway diagrams were originally obtained at KEGG database[^45-47^](#_ENREF_45).

Table S1. The list of drug and xenobiotics metabolizing enzymes and their enzyme classes.

| Genes | Enzyme class |
| --- | --- |
| GSTO2 | 2.5.1.18 |
| GSTA5 | 2.5.1.18 |
| HPGDS | 2.5.1.18 |
| GSTA1 | 2.5.1.18 |
| GSTA2 | 2.5.1.18 |
| GSTA3 | 2.5.1.18 |
| GSTA4 | 2.5.1.18 |
| GSTM1 | 2.5.1.18 |
| GSTM2 | 2.5.1.18 |
| GSTM3 | 2.5.1.18 |
| GSTM4 | 2.5.1.18 |
| GSTM5 | 2.5.1.18 |
| GSTP1 | 2.5.1.18 |
| GSTT1 | 2.5.1.18 |
| GSTT2 | 2.5.1.18 |
| GSTK1 | 2.5.1.18 |
| MGST1 | 2.5.1.18 |
| MGST2 | 2.5.1.18 |
| MGST3 | 2.5.1.18 |
| GSTT2B | 2.5.1.18 |
| GSTO1 | 2.5.1.18 |
| EPHX1 | 3.3.2.9 |
| ADH1A | 1.1.1.1 |
| ADH1B | 1.1.1.1 |
| ADH1C | 1.1.1.1 |
| ADH4 | 1.1.1.1 |
| ADH5 | 1.1.1.1 |
| ADH6 | 1.1.1.1 |
| ADH7 | 1.1.1.1 |
| ALDH3A1 | 1.2.1.5 |
| ALDH1A3 | 1.2.1.5 |
| ALDH3B1 | 1.2.1.5 |
| ALDH3B2 | 1.2.1.5 |
| UGT2B11 | 2.4.1.17 |
| UGT2A1 | 2.4.1.17 |
| UGT2B28 | 2.4.1.17 |
| UGT1A10 | 2.4.1.17 |
| UGT1A8 | 2.4.1.17 |
| UGT1A7 | 2.4.1.17 |
| UGT1A6 | 2.4.1.17 |
| UGT1A5 | 2.4.1.17 |
| UGT1A9 | 2.4.1.17 |
| UGT1A4 | 2.4.1.17 |
| UGT1A1 | 2.4.1.17 |
| UGT1A3 | 2.4.1.17 |
| UGT2A2 | 2.4.1.17 |
| UGT2B4 | 2.4.1.17 |
| UGT2B7 | 2.4.1.17 |
| UGT2B10 | 2.4.1.17 |
| UGT2B15 | 2.4.1.17 |
| UGT2B17 | 2.4.1.17 |
| UGT2A3 | 2.4.1.17 |
| CBR1 | 1.1.1.184 |
| CBR3 | 1.1.1.184 |
| AKR1C1 | 1.3.1.20 |
| AKR7A2 | 1.3.1.20 |
| DHDH | 1.3.1.20 |
| HSD11B1 | 1.1.1.146 |
| CYP4F8 | 1.14.14.1 |
| CYP2A7 | 1.14.14.1 |
| CYP3A7 | 1.14.14.1 |
| CYP2C8 | 1.14.14.1 |
| CYP2C18 | 1.14.14.1 |
| CYP2F1 | 1.14.14.1 |
| CYP2J2 | 1.14.14.1 |
| CYP3A5 | 1.14.14.1 |
| CYP4B1 | 1.14.14.1 |
| CYP4Z1 | 1.14.14.1 |
| CYP4X1 | 1.14.14.1 |
| CYP2S1 | 1.14.14.1 |
| CYP4F11 | 1.14.14.1 |
| CYP3A43 | 1.14.14.1 |
| CYP4F12 | 1.14.14.1 |
| CYP1A1 | 1.14.14.1 |
| CYP2B | 1.14.14.1 |
| CYP1A2 | 1.14.14.1 |
| CYP1B1 | 1.14.14.1 |
| CYP2 | 1.14.14.1 |
| CYP2A6 | 1.14.14.1 |
| CYP2A13 | 1.14.14.1 |
| CYP2C9 | 1.14.14.1 |
| CYP2D6 | 1.14.14.1 |
| CYP2E1 | 1.14.14.1 |
| CYP3A | 1.14.14.1 |
| CYP3A4 | 1.14.14.1 |
| SULT2A1 | 2.8.2.14 |
| FMO1 | 1.14.13.8 |
| FMO2 | 1.14.13.8 |
| FMO3 | 1.14.13.8 |
| FMO4 | 1.14.13.8 |
| FMO5 | 1.14.13.8 |
| MAOA | 1.4.3.4 |
| MAOB | 1.4.3.4 |
| AOX1 | 1.2.3.1 |

| **Table S2.** Significantly differentially expressed (fold change >= 2 and adjusted p-value <= 0.05) DXME genes other than those CYP genes reported in Table 1 in various comparisons. All: overall comparison; ES: early stage (stage I, II); LS: late stage (stage III, IV); LA: luminal A; LB: luminal B; H2: HER2; TN: triple-negative. | | | | | |
| --- | --- | --- | --- | --- | --- |
| **Gene** | **Stage / Subtype of CA vs AA** | **Stage / Subtype of CA vs AS** | | **Stage / Subtype of AA vs AS** |  |
| ADH1A | All, ES | All, LA, ES | | All |  |
| ADH1B |  | All, ES, LA | | All, LA |  |
| ADH1C |  | LA | |  |  |
| AKR1C1 |  | All, ES, LA | | ES, All |  |
| ALDH3A1 | All, ES |  | | All, ES |  |
| AOX1 | H2 |  | |  |  |
| DHDH | All, ES, LS, LA, TN |  | | All, ES |  |
| FMO2 |  | All, ES, LA | | All, ES |  |
| FMO3 |  |  | | TN |  |
| FMO4 |  | LS | |  |  |
| FMO5 | LS |  | |  |  |
| GSTA1 | LS |  | | ES |  |
| GSTM1 | H2 |  | | All |  |
| GSTM2 | H2 |  | | All, ES |  |
| GSTM5 |  | All, ES, LA | | LA |  |
| GSTT2 | LA, LB |  | | All |  |
| HSD11B1 | H2 |  | |  |  |
| UGT1A6 | LS |  | |  |  |
| UGT2B10 | LA |  | |  |  |
| UGT2B11 | All, ES, LA |  | | All |  |
| UGT2B28 | LA |  | | All, LS |  |
| UGT2B4 | All, LA, LS |  | |  |  |
|  | | |  | | |

**Table S3.** The expressions of ADH genes.

| Genes | All BRCA | Adjacent normal | Caucasian American | African American | Asian American |
| --- | --- | --- | --- | --- | --- |
| ADH1A | 0.46 | 15.19 | 0.53 | 0.23 | 0.12 |
| ADH1B | 52.70 | 1649.03 | 61.46 | 29.79 | 16.76 |
| ADH1C | 1.61 | 17.30 | 1.70 | 2.05 | 1.28 |
| ADH4 | 0.084 | 1.74 | 0.09 | 0.08 | 0.11 |
| ADH5 | 104 | 194 | 106 | 100 | 102 |
| ADH6 | 0.0611 | 0.125 | 0.0669 | 0.0542 | 0.0555 |
| ADH7 | 0.349 | 0.506 | 0.475 | 0.165 | 0.00246 |

|  |
| --- |
|  |

**Table S4.** The summary of clinical information of the cohort in this study.

| Clinical Information | Caucasian American | African American | Asian American |
| --- | --- | --- | --- |
| Number of patients | 728 | 143 | 57 |
| Age | | | |
| 0-49 | 197 | 48 | 23 |
| 50-64 | 311 | 55 | 26 |
| 65- | 220 | 40 | 8 |
| Stages | | | |
| Stage 0 | 1 | 1 | 0 |
| Stage I | 134 | 27 | 4 |
| Stage II | 409 | 81 | 41 |
| Stage III | 165 | 28 | 12 |
| Stage IV | 11 | 2 | 0 |
| Unknown | 8 | 4 | 0 |
| Subtypes | | | |
| Luminal A | 298 | 34 | 21 |
| Luminal B | 77 | 4 | 5 |
| HER2 | 19 | 4 | 7 |
| Triple-negative | 67 | 26 | 8 |
| Unknown | 267 | 75 | 16 |
| Vital status | | | |
| Alive | 648 | 126 | 56 |
| Dead | 80 | 17 | 1 |

**Tumoral expression of drug and xenobiotic metabolizing enzymes in breast cancer patients of different ethnicities with implications to personalized medicine**

**Additional figures**

**Volcano plots for varies comparisons of DXME expression. The titles of the figures are self-descriptive.**

**
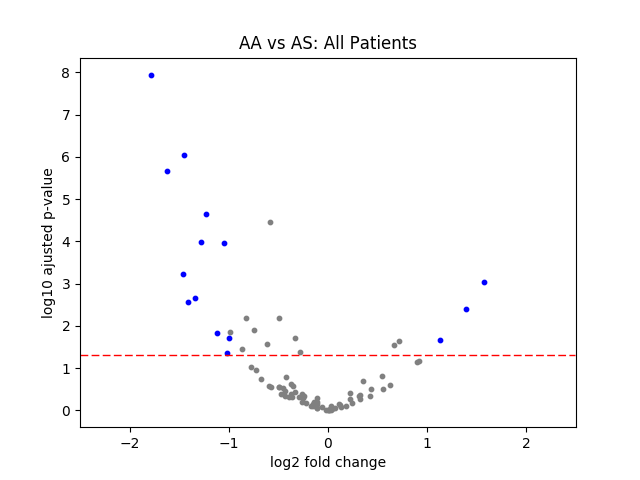

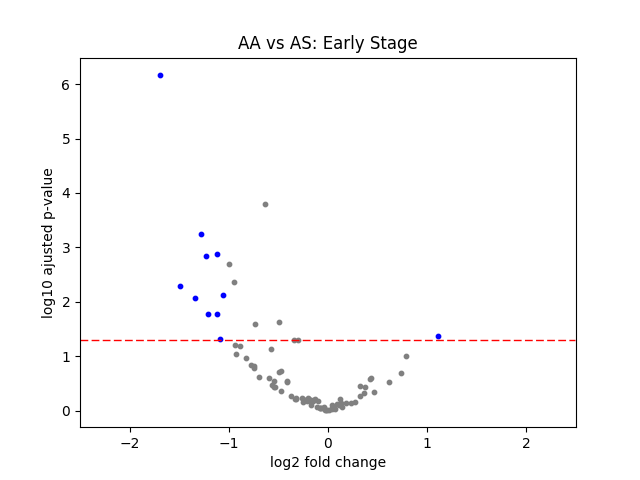

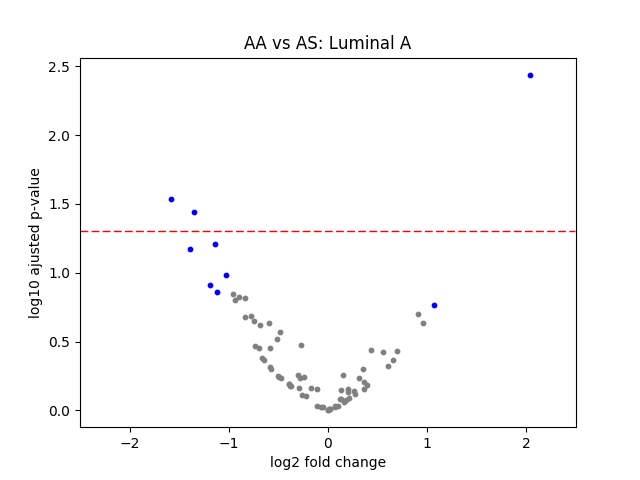

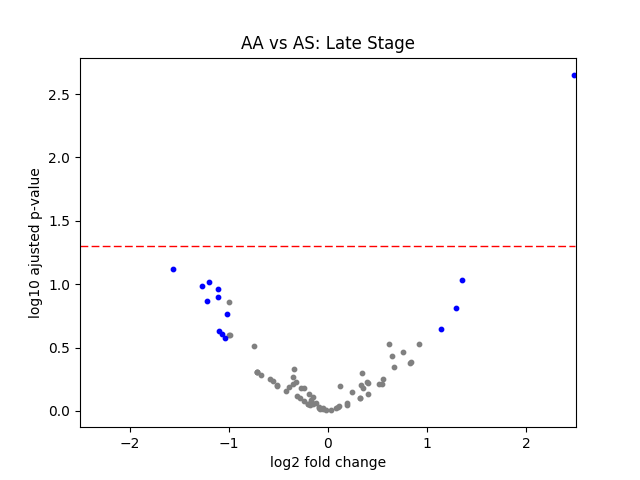

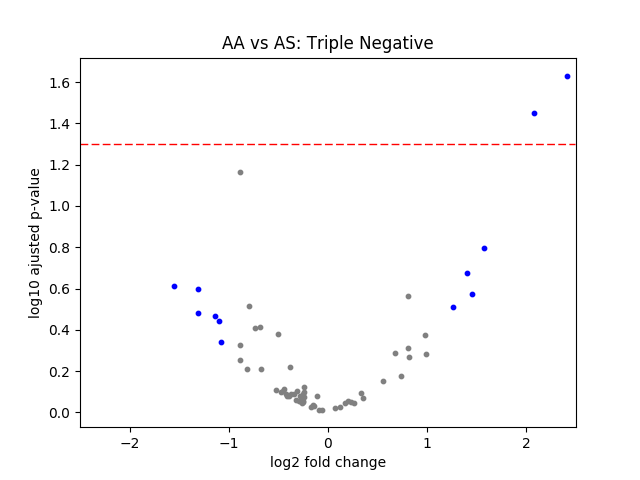

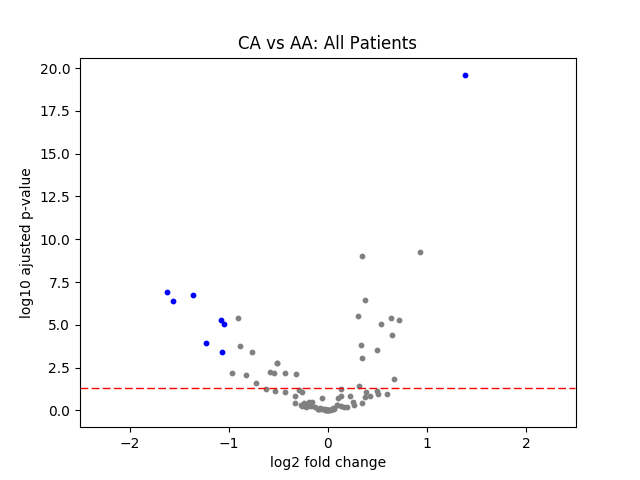

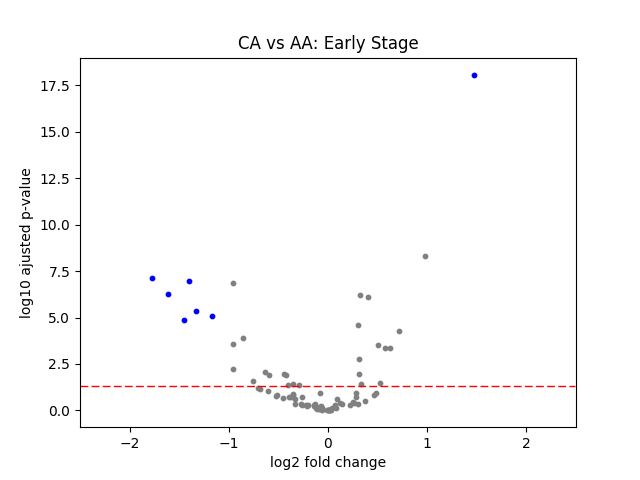

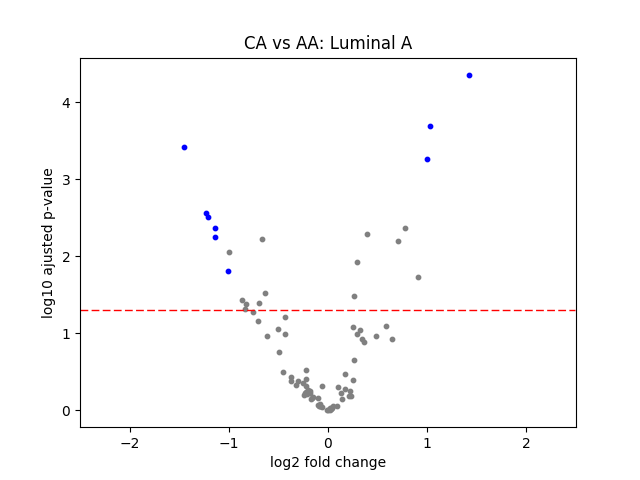

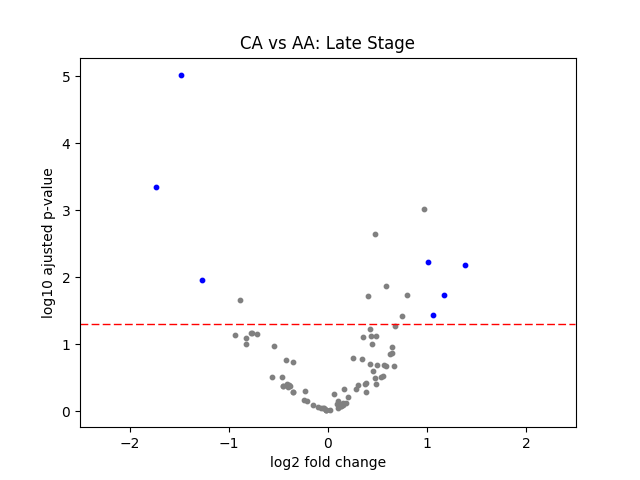

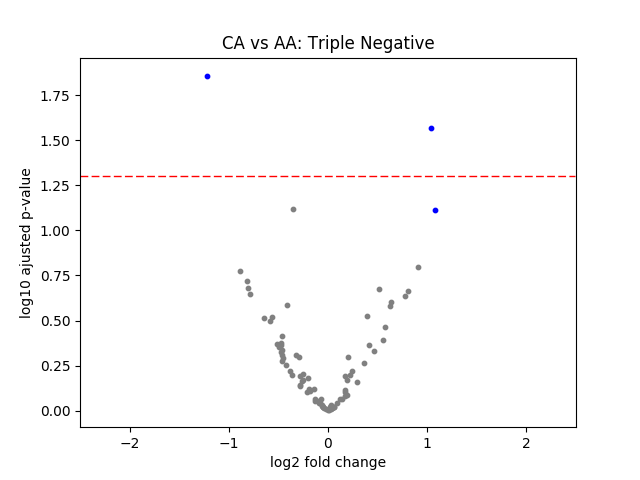

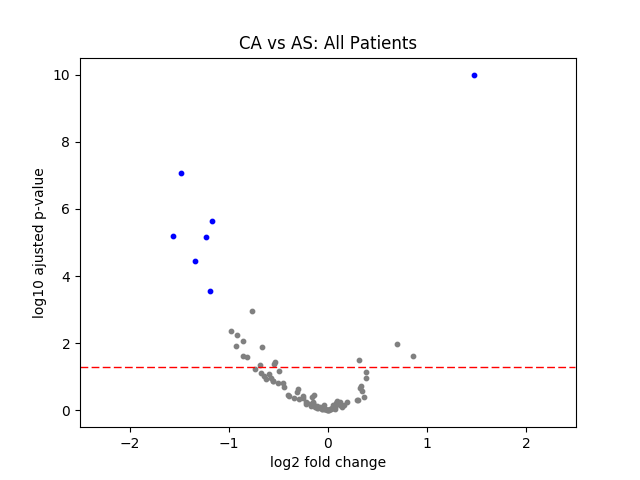

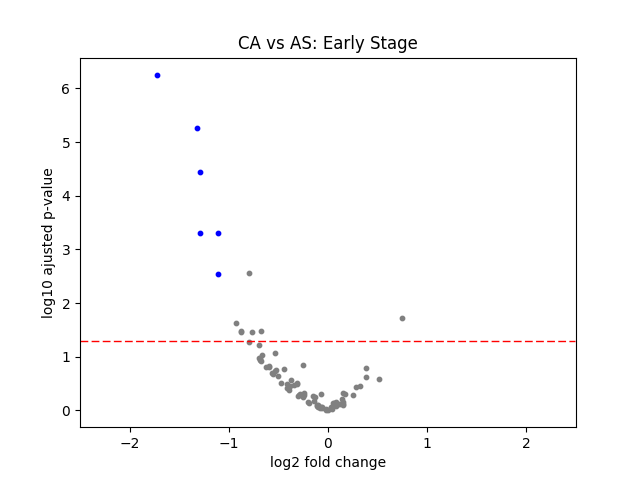

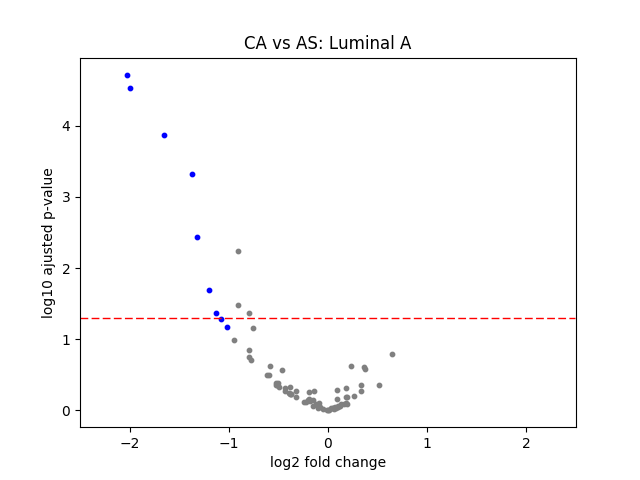

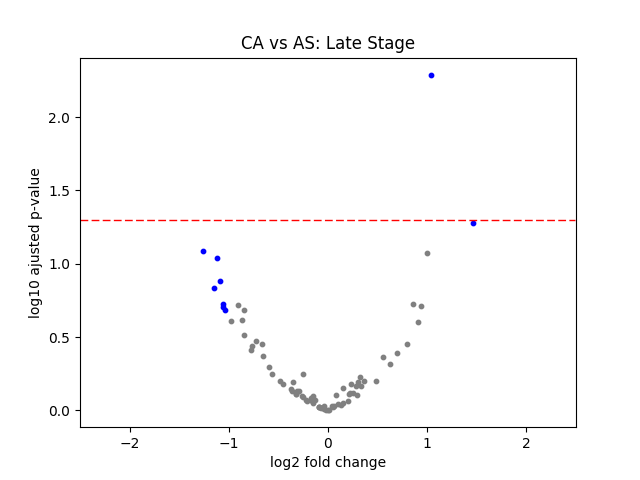

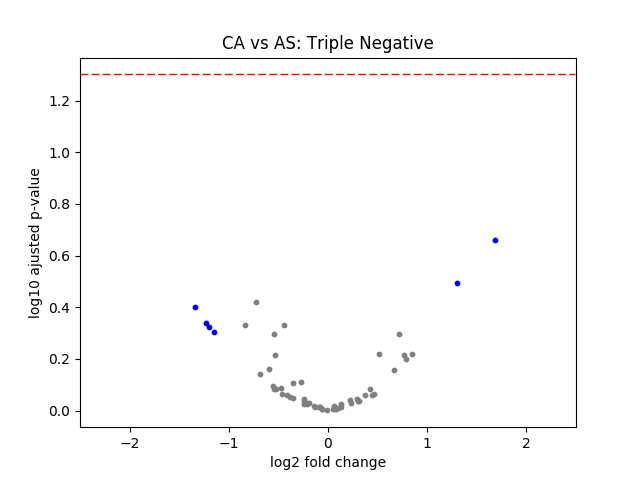
**

**Heatmaps for varies comparisons.**

**
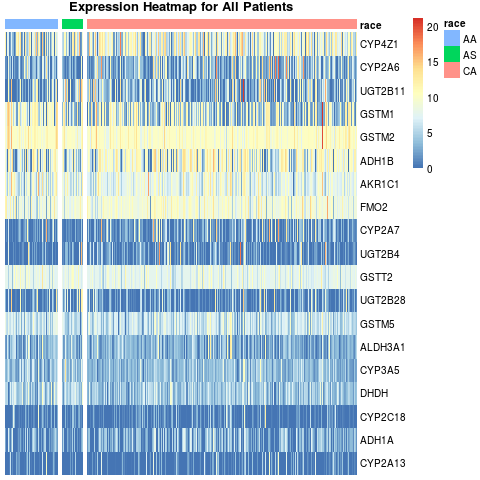
**


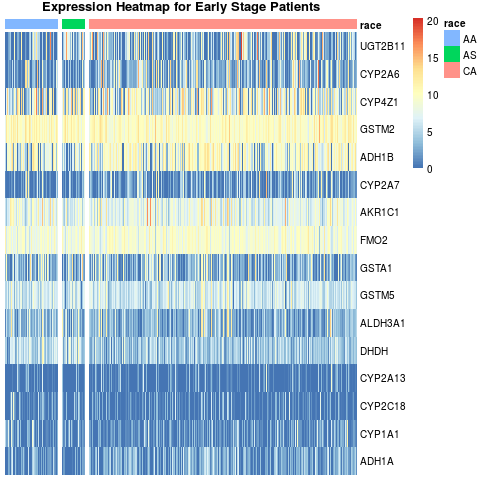

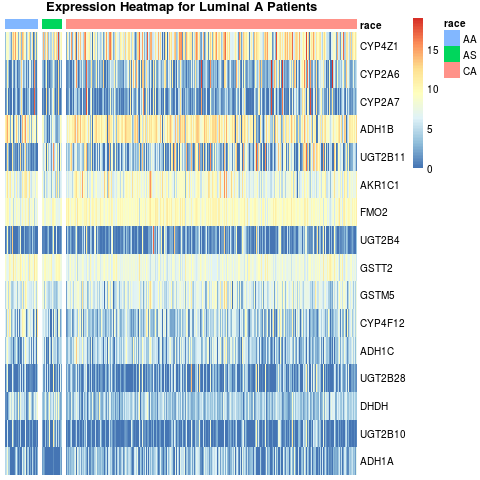

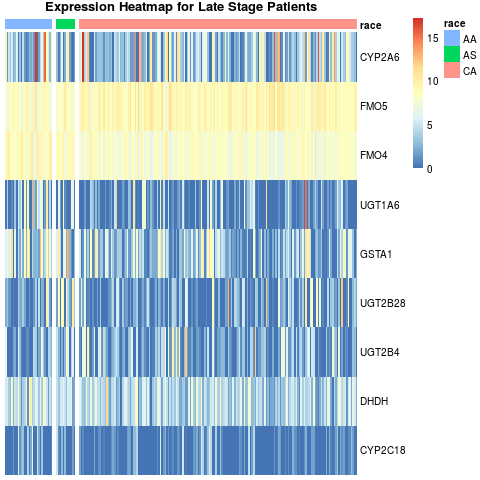

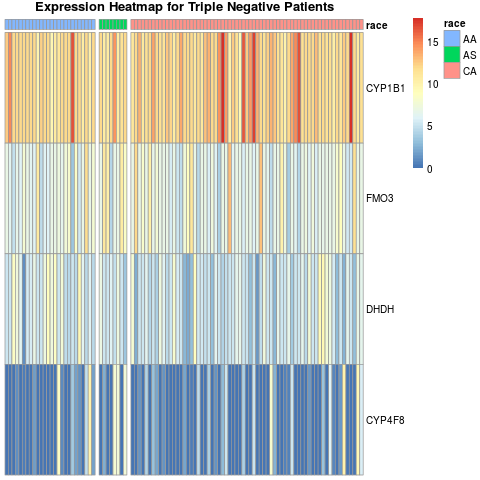


**PCA plots.**


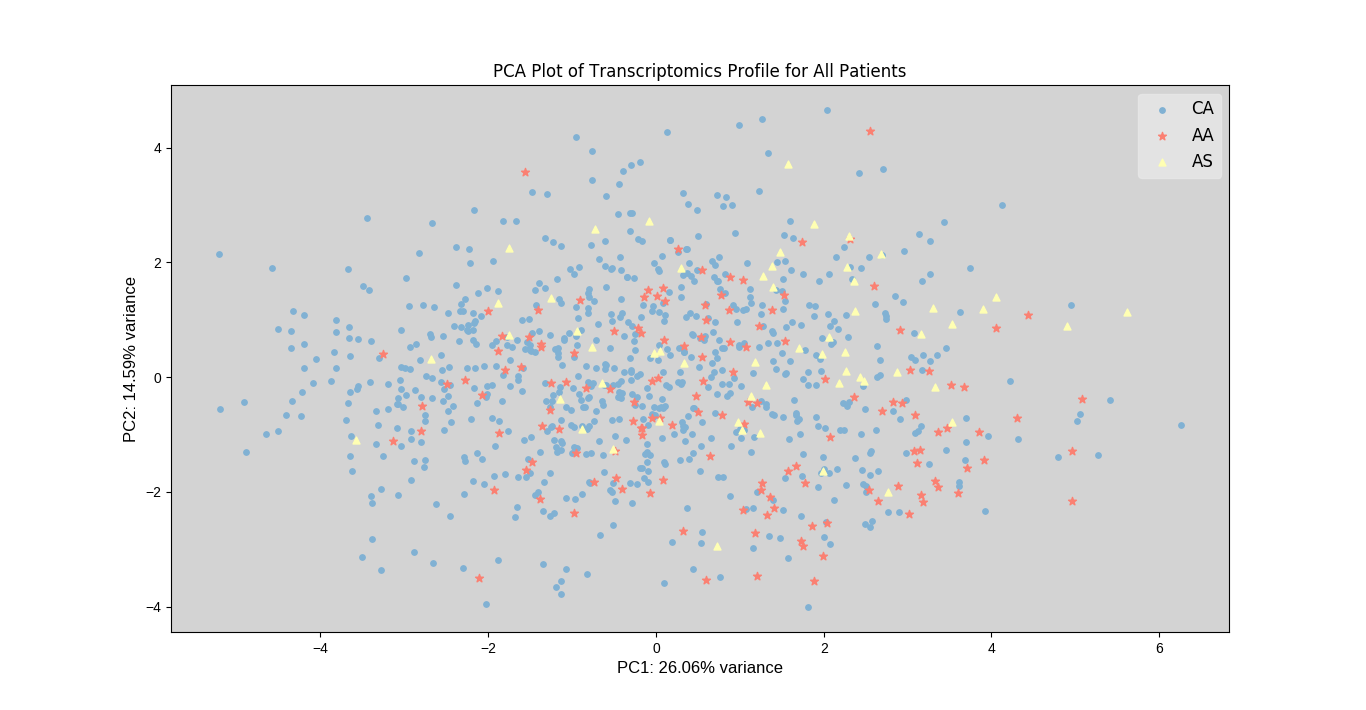

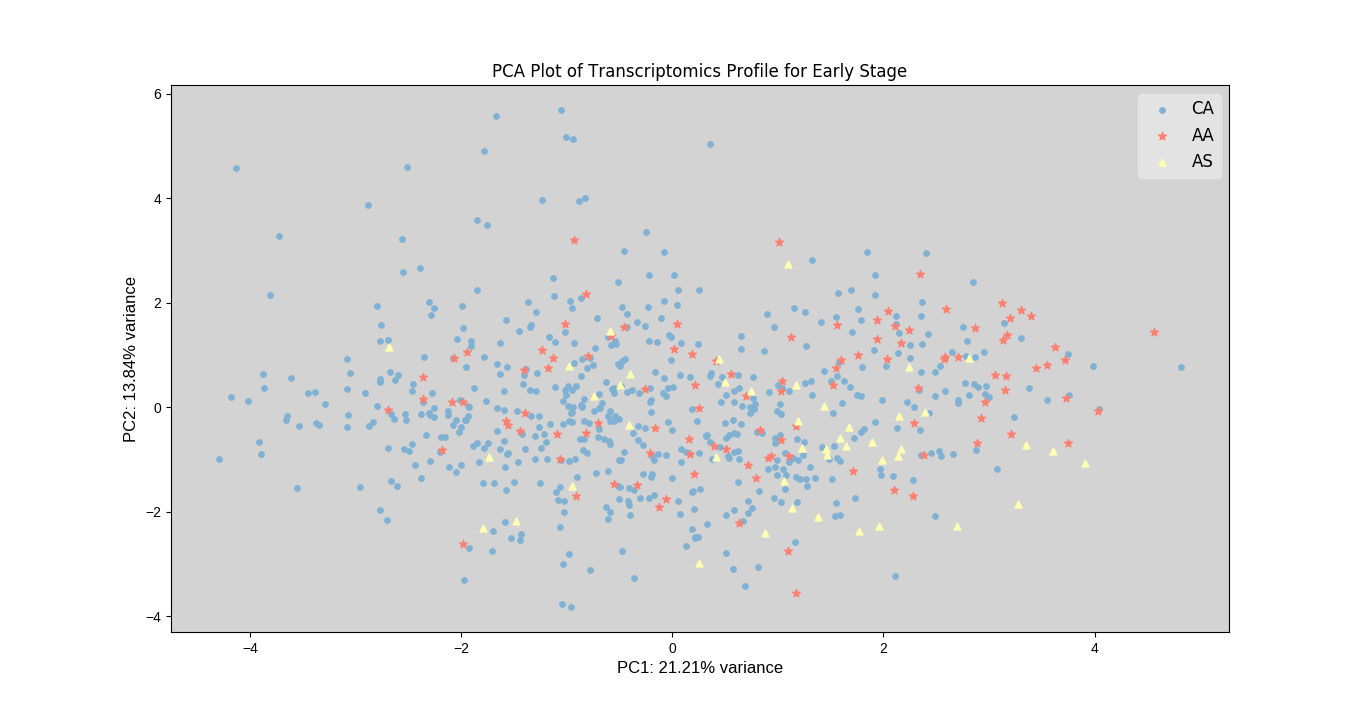

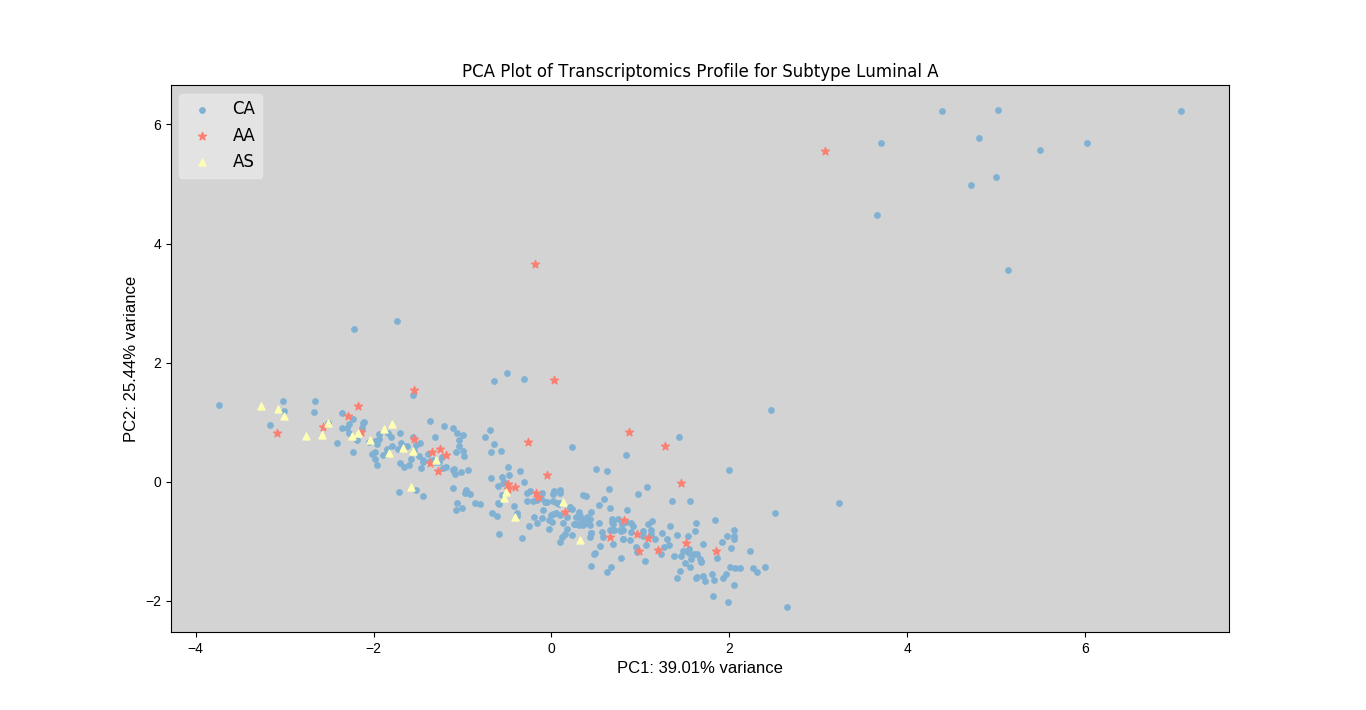

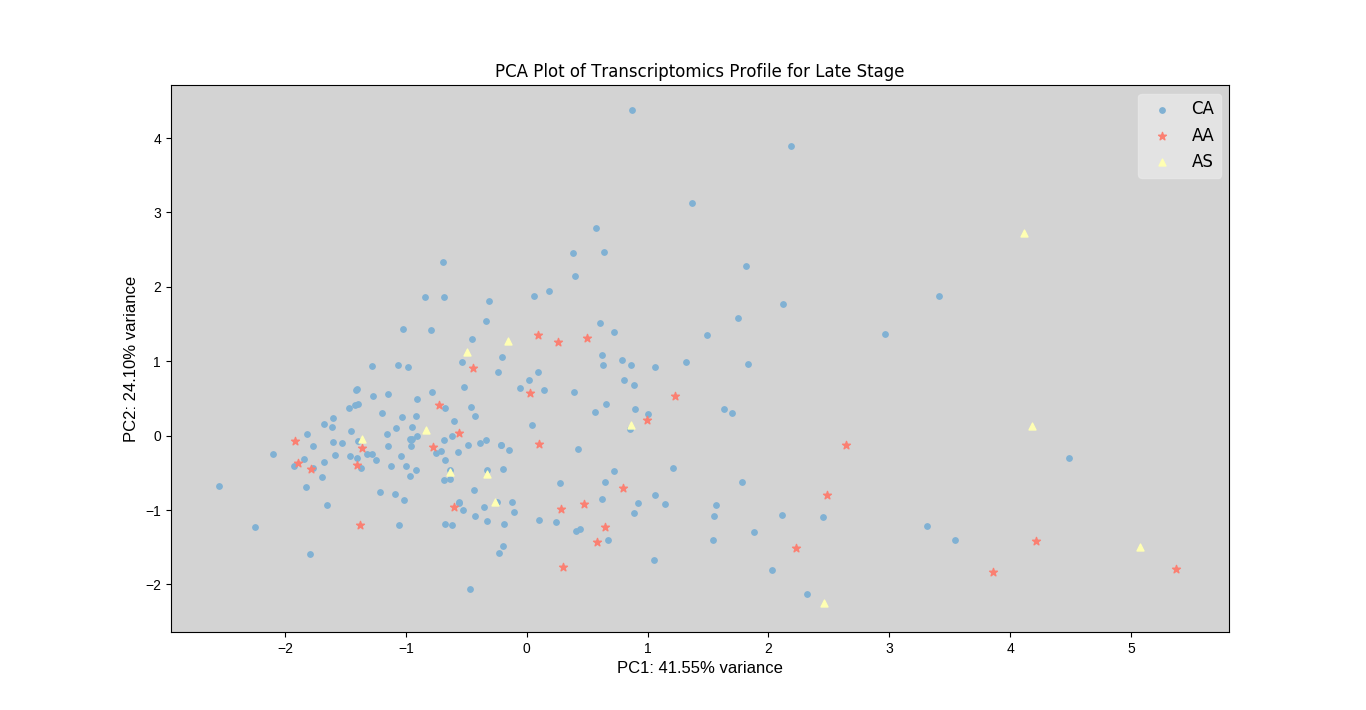

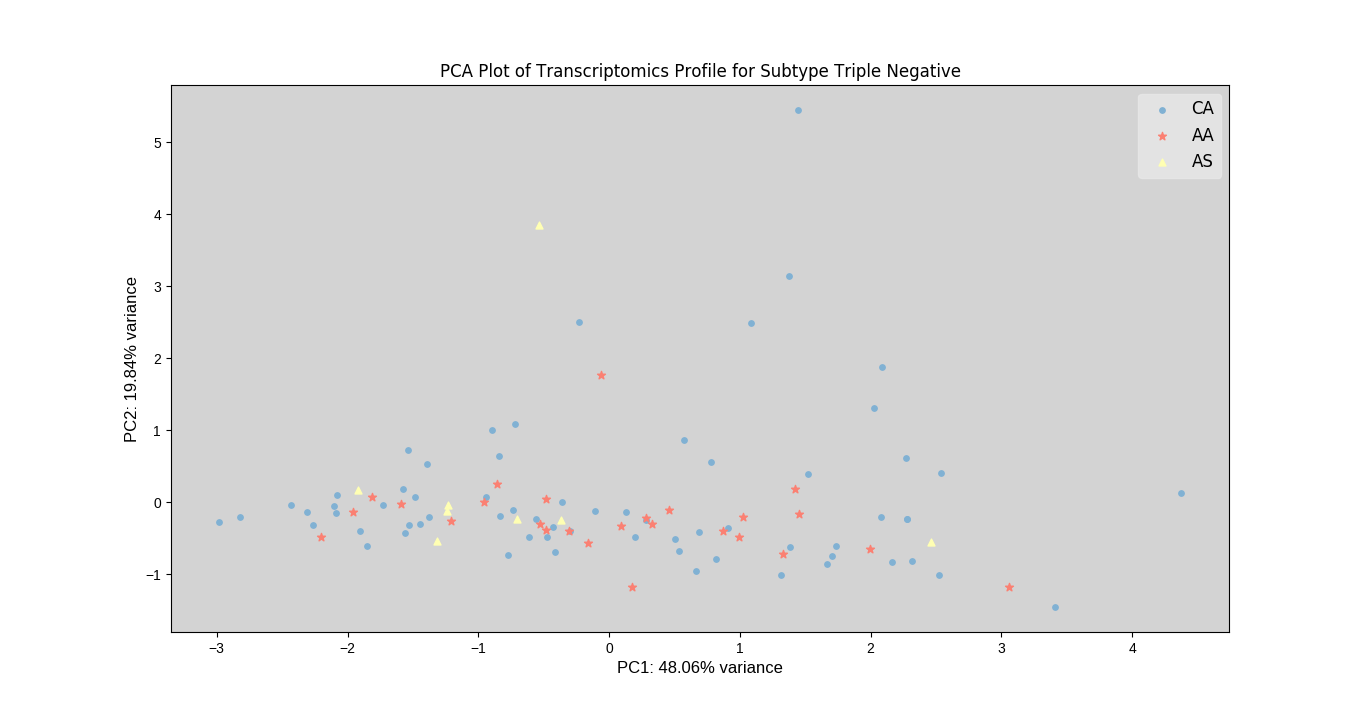


**Bar chart.**


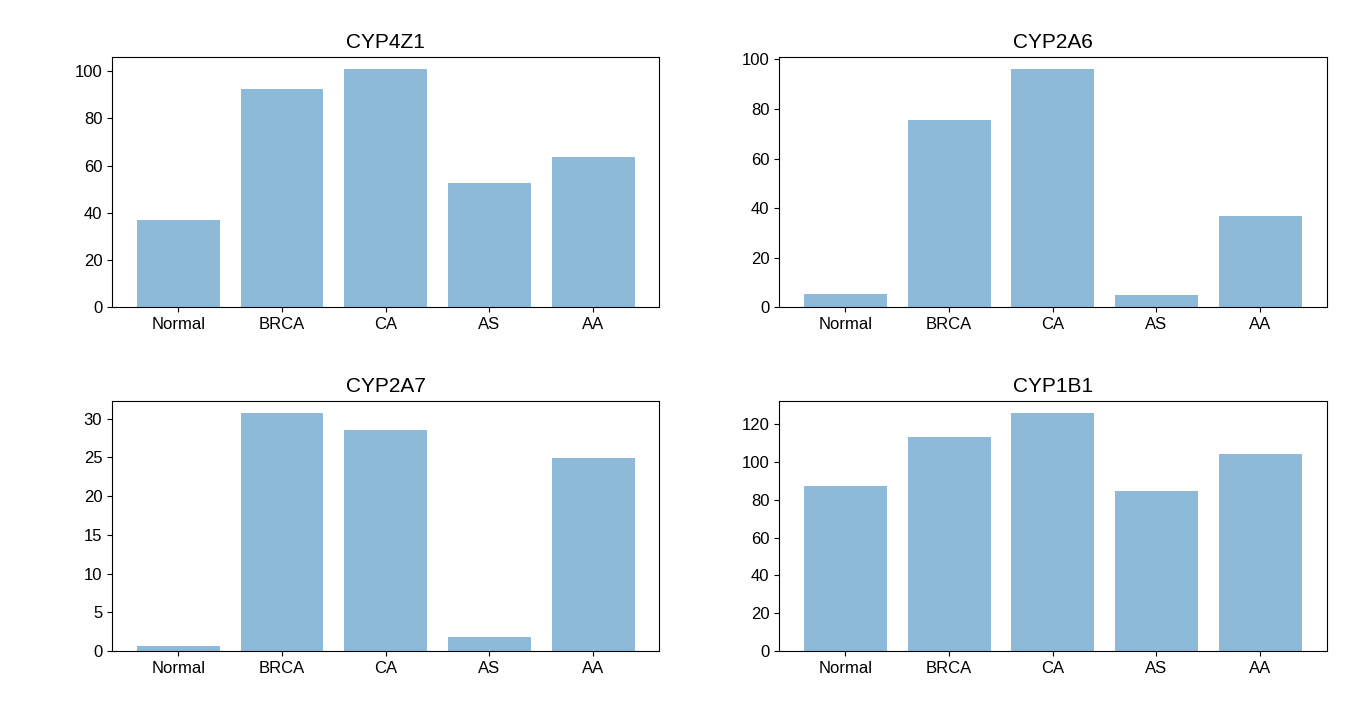

Supplement: Supplementary file 1 — Supplementary Material 1 [file 41598_2017_4250_MOESM1_ESM.doc]
